# Stochastic interventional approach to assessing immune correlates of protection: Application to the COVE messenger RNA-1273 vaccine trial

**DOI:** 10.1016/j.ijid.2023.09.012

**Published:** 2023-10-25

**Authors:** Nima S. Hejazi, Xiaoying Shen, Lindsay N. Carpp, David Benkeser, Dean Follmann, Holly E. Janes, Lindsey R. Baden, Hana M. El Sahly, Weiping Deng, Honghong Zhou, Brett Leav, David C. Montefiori, Peter B. Gilbert

**Affiliations:** 1 Department of Biostatistics, T.H. Chan School of Public Health, Harvard University, Boston, USA; 2 Department of Surgery and Duke Human Vaccine Institute, Duke University Medical Center, Durham, USA; 3 Vaccine and Infectious Disease Division, Fred Hutchinson Cancer Center, Seattle, USA; 4 Department of Biostatistics and Bioinformatics, Rollins School of Public Health, Emory University, Atlanta, USA; 5 Biostatistics Research Branch, National Institute of Allergy and Infectious Diseases, National Institutes of Health, Bethesda, USA; 6 Division of Public Health Sciences, Fred Hutchinson Cancer Center, Seattle, USA; 7 Department of Biostatistics, University of Washington, Seattle, USA; 8 Division of Infectious Diseases, Harvard Medical School, Brigham and Women’s Hospital, Boston, USA; 9 Department of Molecular Virology and Microbiology, Baylor College of Medicine, Houston, USA; 10 Infectious Disease Development, Moderna, Inc., Cambridge, USA

**Keywords:** Correlate of protection, Neutralizing antibody titer, Phase III trial, Stochastic interventional vaccine efficacy analysis

## Abstract

**Background:**

Stochastic interventional vaccine efficacy (SVE) analysis is a new approach to correlate of protection (CoP) analysis of a phase III trial that estimates how vaccine efficacy (VE) would change under hypothetical shifts of an immune marker.

**Methods:**

We applied nonparametric SVE methodology to the COVE trial of messenger RNA-1273 vs placebo to evaluate post-dose 2 pseudovirus neutralizing antibody (nAb) titer against the D614G strain as a CoP against COVID-19. Secondly, we evaluated the ability of these results to predict VE against variants based on shifts of geometric mean titers to variants vs D614G. Prediction accuracy was evaluated by 13 validation studies, including 12 test-negative designs.

**Results:**

SVE analysis of COVE supported post-dose 2 D614G titer as a CoP: estimated VE ranged from 66.9% (95% confidence interval: 36.2, 82.8%) to 99.3% (99.1, 99.4%) at 10-fold decreased or increased titer shifts, respectively. The SVE estimates only weakly predicted variant-specific VE estimates (concordance correlation coefficient 0.062 for post 2-dose VE).

**Conclusion:**

SVE analysis of COVE supports nAb titer as a CoP for messenger RNA vaccines. Predicting variant-specific VE proved difficult due to many limitations. Greater anti-Omicron titers may be needed for high-level protection against Omicron vs anti-D614G titers needed for high-level protection against pre-Omicron COVID-19.

## Introduction

Stochastic interventional vaccine efficacy (SVE) analysis is a new approach to the assessment of immune correlates of protection (CoPs) based on a phase III vaccine efficacy (VE) trial dataset, recently published in the statistical literature [1]. The SVE method is a causal inference-based technique for investigating how VE in an efficacy trial would be expected to shift based on hypothetical perturbations of an immune marker measured at a fixed time point post-vaccination. The degree of hypothetical perturbation is selected by the analyst, facilitating exploration of the immune marker-VE dose-response relationship. This technology transfer article introduces the SVE framework to a non-statistician vaccine development audience, with application to the COVE trial. To the best of our knowledge, this is the first application of the SVE approach to a phase III VE dataset to assess an immune CoP.

Previous immune correlates analyses of phase III VE trials by the US government’s COVID-19 Vaccine Correlates of Protection Program [2–5] used controlled vaccine efficacy (CVE) analysis [6], which assesses how assignment of all participants to vaccination and to a fixed immune marker value (a deterministic intervention) impacts VE (vs placebo). SVE analysis instead considers a stochastic intervention, which specifies that the immune marker of vaccine recipients is shifted from its observed value by a user-supplied shifting function. By framing the hypothetical intervention as a change (or shift) *relative* to observed immune marker levels of individual vaccine recipients, the SVE approach delineates how VE would be expected to change upon perturbation of an immune marker. The SVE approach recognizes immune response heterogeneity by considering only relative, individual-level shifts while remaining potentially informative for refined vaccines designed to elicit higher immune marker values.

Directly estimating VE against symptomatic infection with a given SARS-CoV-2 variant using “gold standard” data from a randomized, placebo-controlled efficacy trial (RCT) can only be done for variants circulating during the same time frame and at the same locations where the trial was conducted. The COVE phase III trial of the messenger RNA (mRNA)-1273 vaccine vs placebo (NCT04470427) was conducted early in the pandemic in geographic regions where only ancestral lineage (i.e., B.1/B.1.2 lineage, with characteristic mutation D614G vs the index strain, NC_045512.2) viruses and minor genetic drift variants circulated [7]. Estimated VE against COVID-19 illness from 14 days post-dose 2 through completion of the blinded phase (median follow-up 5.3 months) was 93.2% (95% confidence interval [CI]: 91.0, 94.8%) [7]. Pajon et al. [8] reported VE estimates against COVID-19 caused by epsilon B.1.427 or B.1.429, epsilon B.1.429, and epsilon or gamma. In the phase II/III trial of the BNT162b2 vaccine, estimated VE against COVID-19 illness from 7 days post-dose 2 through completion of the blinded phase (6 months of follow-up) was 91.3% (95% CI: 89.0, 93.2%) [9]. In South Africa, where the beta variant predominated, estimated BNT162b2 VE was 100% (95% CI: 53.5, 100%) [9]. Without additional RCT data, test-negative design (TND) and case-control studies have also been used to estimate effectiveness of the mRNA-1273 and BNT162b2 vaccines against SARS-CoV-2 infection and/or COVID-19 illness caused by variants [10–20].

A validated immune CoP [21] is a biomarker that can be used to reliably infer the degree of vaccine protection against a clinical endpoint and applied to establish a basis of vaccine approval (e.g., variant-adapted vaccines) when RCT data are unavailable. A body of evidence supports nAb titer against the index or D614G strain as a CoP for COVID-19 vaccines (Supplementary Text and [22]). These results provide a basis for nAb titers against contemporary SARS-CoV-2 strains for recipients of a variant-adapted vaccine to be used for its authorization or approval, typically through immunobridging studies. In addition, a nAb titer CoP can be used for estimating VE against variants by incorporating data on nAb titers to the variants. Moreover, variant-adapted boosters that better-match newer circulating strains may provide higher protection (vs the original vaccine-strain booster) against symptomatic COVID-19 caused by the newer strains [23].

Using a population-level approach, Cromer et al. [24] modeled how well nAb titers against variants could be used to predict VE against those variants, reporting good prediction based on the mean decrease in nAb titer against the variant strain vs the ancestral strain. Here, we apply the SVE approach using individual-level data from a single RCT, combined with data on nAb titers against variants. Our objectives are, first, to show how the SVE framework helps define nAb titer as a correlate of protection against ancestral strain COVID-19, and, secondly, to evaluate how well the SVE framework can be used to predict two-dose and three-dose VE against variant-specific symptomatic infection based on concordance analysis with direct estimates of VE obtained from TND studies and one RCT.

## Methods

### Neutralizing antibody assay

Neutralizing antibodies against SARS-CoV-2 spike-pseudotyped virus were measured using 293T/ACE2 cells as described [2]. Using the World Health Organization anti-SARS-CoV-2 Immunoglobulin International Standard (20/136), pseudovirus neutralizing antibody 50% inhibitory dilution (PsV-nAb ID50) titers against the D614G strain were converted to IU50/ml using the conversion factor of 0.242. ID50 titers against other variants were also multiplied by 0.242 to aid readout comparability across strains.

### Stochastic interventional vaccine efficacy analysis

The SVE approach estimates the counterfactual VE in a RCT under relative, individual-level perturbations of an immune marker measured at a fixed post-vaccination time-point. The stochastic intervention considers a perturbation δ to the observed immune marker S for vaccine recipients, shifting S to generate a counterfactual immune response S + δ, for an analyst-specified value δ. Under this hypothetical intervention, with A the indicator of receiving vaccine as opposed to placebo and X a set of baseline adjustment covariates (minority demographics indicator, high risk-of-infection indicator, baseline risk score), and under causal inference assumptions noted below, the counterfactual risk of infection or disease outcome (event Y = 1) can be expressed as $\text{E[P(Y }=\;1\;\left|\text{ S }=\text{ S }+\text{ δ, A }=\;1\text{, X }=\text{ x) }\right|\text{ A }=\;1\text{, X]}$, where counterfactual immune response S + δ is higher (for δ > 0) or lower (for δ < 0) than the observed immune response S. The SVE, itself a function of δ, equals

$$\text{SVE(δ) = }\left\{1\;-\;\frac{\text{E[P(Y }=\;1\;\left|\text{ S }=\text{ S }+\text{ δ, A }=\;1\text{, X }=\text{ x) }\right|\text{ A }=\;1\text{, X]}}{\text{E[P(Y }=\;1|\text{A }=\;0\text{, X = x)|X]}}\right\}\;\times \;100\text{\%,}$$

where the term in the denominator is the placebo arm risk (E[] and P[] are expectation and probability operators). Intuitively, the SVE, which depends on the magnitude of the perturbation δ, is one minus a proportion of the mean risk for vaccine recipients assigned the given perturbation and the mean risk for placebo recipients. When the SVE curve increases/decreases with the degree of perturbation, it indicates that increasing/decreasing immune marker levels increases/decreases vaccine efficacy.

When δ = 0, indicating that the immune marker S is not modified, SVE(δ) equals the overall VE, readily estimable from randomization to vaccine vs placebo. The SVE approach formalizes a framework for considering how hypothetical changes (represented by δ) to an immune marker’s level impact VE. Using phase I study data collected on the PsV-nAb ID50 titer against SARS-CoV-2 variants, we use this anchoring in our SVE analysis by setting δ as the mean difference in response between said variants and the D614G virus. Evaluation of SVE(δ) across multiple choices of δ describes how VE is expected to change under different magnitude shifts in an immune marker. For example, when δ is measured as $\text{lo}{\text{g}}_{10}$ fold-change in geometric mean PsV-nAb ID50, comparison of SVE(δ = 0) to SVE(δ = 1) informs on how much VE would be expected to change with a refined version of the vaccine regimen that increased PsV-nAb ID50 titer by an order of magnitude. Repeating this process across several choices of δ, then, “traces out” a curve of SVE(δ) vs δ [1]. We perform such an analysis, interpreting δ based on differences in PsV-nAb ID50 titers against variants relative to against D614G. The assumptions made in applying the SVE approach to estimate post-dose 2 VE against the COVID-19 primary endpoint of the COVE study under a given mean shift in log10 PsV-nAb ID50 titer are detailed in the Supplementary Material.

The estimated curve SVE(δ) provides a way to predict VE against COVID-19 caused by SARS-CoV-2 variants other than the ancestral virus. Specifically, for a given SARS-CoV-2 variant, let δ be the difference in mean $\text{lo}{\text{g}}_{10}$ PsV-nAb ID50 titer against the variant compared to against the D614G strain. The estimate of VE at this shift value provides an estimate of VE against the variant-specific COVID-19. How can this estimate be interpreted? Envisage a ‘hypothetical variant trial’ that is the same as the actual COVE study in all respects except that we suppose that the variant had been the sole circulating viral lineage during the trial (instead of the ancestral lineage) and that its placebo-arm incidence of COVID-19 was the same as the placebo-arm incidence of ancestral-virus COVID-19 in the actual COVE study (this is a placebo arm *scenario* for interpretation of results, not an assumption). Then, the estimate of VE against the variant is interpreted as the VE that would have resulted in the hypothetical variant trial under a ‘variant-invariant CoP model’ assumption (terminology from Jerry Sadoff, personal communication), which can be posited as follows for use of the SVE method: the mean counterfactual risk of D614G-virus-specific COVID-19 when assigning all participants to two doses of the mRNA-1273 vaccine and the δ mean shift in anti-D614G $\text{lo}{\text{g}}_{10}$ PsV-nAb ID50 titer in the COVE trial equals the mean risk of variant-specific COVID-19 when assigning all participants to two doses of the mRNA-1273 vaccine (without a shift) in the two-dose hypothetical variant trial. In other words, the ancestral lineage-specific VE at a particular titer level of antibodies against ancestral lineage virus is the same as variant-specific VE against a given variant at the same titer level of antibodies against that variant. We also consider prediction of post-dose 3 VE (vs three doses of placebo) against COVID-19 with a specific variant (see Supplementary Material for assumptions).

### Inclusion of vaccine efficacy/effectiveness estimates in the validation analysis

The literature search process and criteria for inclusion of a variant-specific VE estimate in the validation analysis are described in the Supplementary Material. In brief, the validation analysis considered all available studies of an mRNA vaccine that measured SARS-CoV-2 sequences from COVID-19 cases, estimated lineage-specific vaccine effectiveness (vs unvaccinated), and met other criteria for feasibility of comparing results with those from the COVE study. Twelve TND studies [10–20,25] and one RCT [8] qualified and were used as comparators.

## Results

### Neutralizing antibody titers against SARS-CoV-2 variants

**Fig. 1.**
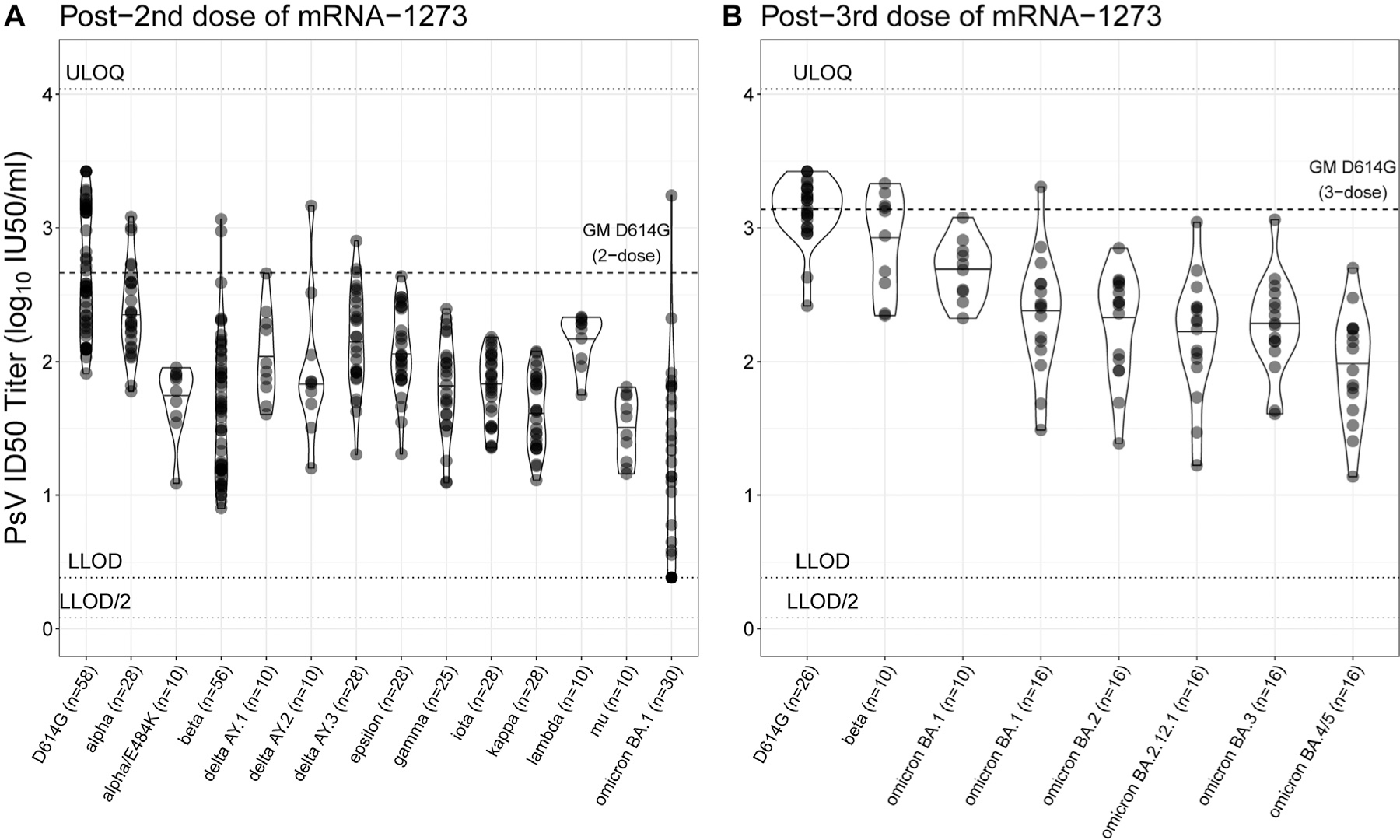
PsV-nAb ID50 titers of serum from recipients of (a) two or (b) three doses of mRNA-1273 against different SARS-CoV-2 variants. PsV-nAb ID50 titers of serum samples drawn 4 weeks after two or three doses of the mRNA-1273 vaccine (clinical studies detailed information in Table 1) were assessed against variant spike-pseudotyped viruses. The dotted horizontal line represents the geometric mean PsV-nAb ID50 against the ancestral strain D614G. Each dot represents one participant. Numbers in parentheses after each variant name are the sample sizes. Supplementary Table 2 provides summary statistics of the geometric mean PsV-nAb ID50 titers. GM, geometric mean; LLOD, lower limit of detection; mRNA, messenger RNA; PsV-nAB, pseudovirus neutralizing antibody; ULOQ, upper limit of quantitation. IU50/ml, International Units 50/ml (calibrated to the D614G strain World Health Organization International Standard 20/136 for anti-SARS-CoV-2 immunoglobulin).

Table 1 describes the studies from which mRNA-1273 vaccine recipient serum samples were obtained for nAb assays and the spike variant assayed (Supplementary Table 1). nAb data were measured against 18 different variants, with data from between 10 and 58 participants per variant. Fig. 1a and b show the distribution of PsV-nAb ID50 titers against each variant, for two-dose and three-dose vaccine recipients, respectively; Supplementary Table 2 provides summary statistics of the geometric mean PsV-nAb ID50 titers. In general PsV-nAb ID50 geometric mean titer (GMT) against each variant was lower than corresponding dose-specific GMT against D614G. GMT generally decreased with chronological time of variant emergence defined by date of World Health Organization-Variant Classification, such that antigenic distance increased with successive variant emergence.

### Stochastic interventional vaccine efficacy correlates of protection analysis of COVE

**Fig. 2.**
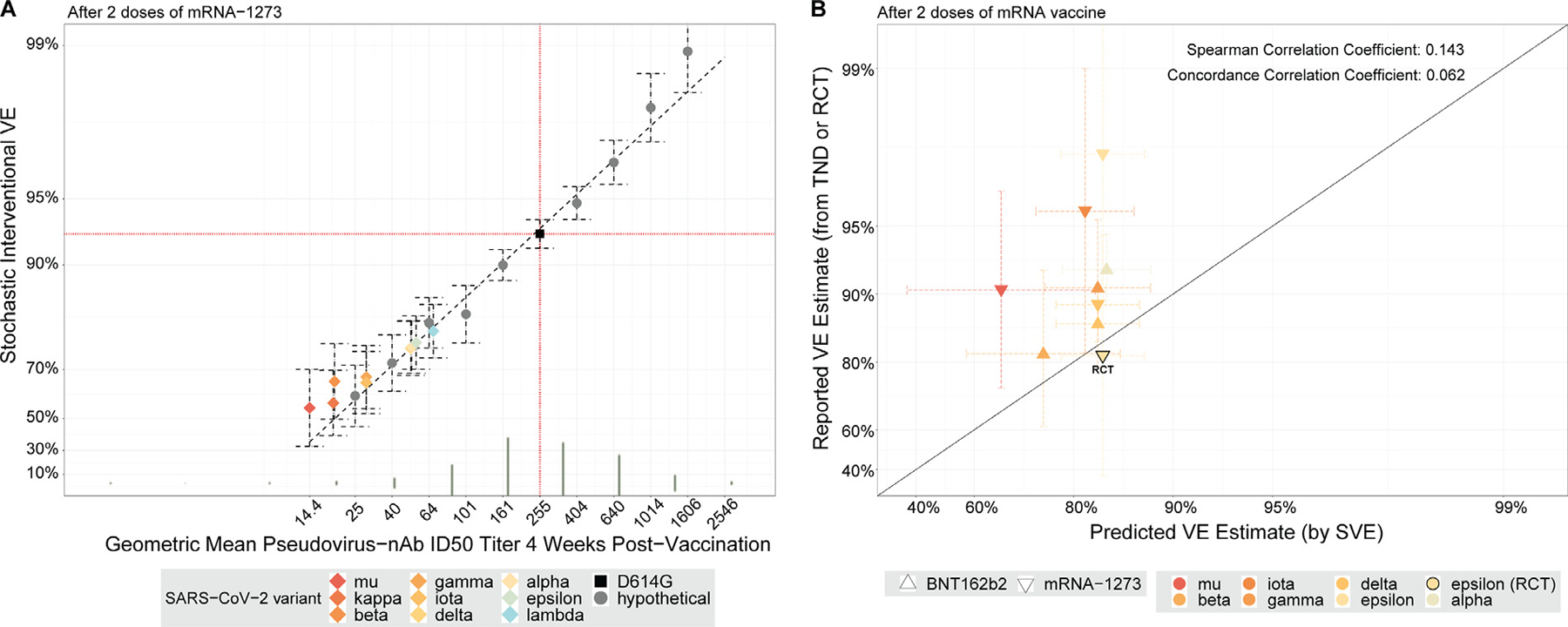
For two-dose mRNA-1273 recipients, stochastic interventional vaccine efficacy estimates under hypothetical shifts of neutralizing antibody titer, with application to predict vaccine efficacy against different SARS-CoV-2 variants, with validation analysis from external studies of two vaccine doses. (a) Y-axis: Estimated vaccine efficacy against virologically confirmed, symptomatic COVID-19 disease for a vaccine that elicits geometric mean 4-week post dose 2 PsV-nAb ID50 titer against the ancestral strain with the value indicated on the x-axis, estimated using the method in Hejazi et al. [1]. ID50 titer = IU/ml because ancestral strain titers were calibrated to the World Health Organization International Standard 20/136 D614G strain for anti-SARS-CoV-2 immunoglobulin. The circles, squares, and diamonds are the SVE estimates, with 95% confidence intervals (dashed lines), for a vaccine that hypothetically shifts each vaccine recipient’s $\text{lo}{\text{g}}_{10}$ nAb ID50 titer by a constant amount (for the black square, δ = 0) from their observed value against D614G that yields the given geometric mean D57 PsV-nAb ID50 titer against the designated variant (see Methods). For the diamonds, antigen panel data (Table 1) were used to estimate the actual mean shift in $\text{lo}{\text{g}}_{10}$ PsV-nAb ID50 titer for a response against the D614G strain as opposed to against the designated SARS-CoV-2 variant. SVE estimates are reported for the follow-up period 7 through 100 days post day 57. The diagonal dashed line gives a linear model summary of how SVE(δ) is expected to change with $\text{lo}{\text{g}}_{10}$ geometric mean titer shifts (δ) (see the Supplementary Text for further details). The vertical green bars indicate the observed distribution of PsV-nAb ID50 titer as measured from participants post dose 2 in the COVE trial. (b) Comparison of VE estimates against SARS-CoV-2 infection or symptomatic COVID-19 caused by different SARS-CoV-2 variants obtained in a phase III RCT or in TND studies of index-strain COVID-19 mRNA vaccines compared to SVE estimates in panel A. Estimates are for after two doses of mRNA vaccine (mRNA-1273 and/or BNT162b2) vs placebo. The reported VE estimate for each plotted symbol corresponds to the average (taken on the log scale and back-transformed to the VE scale) of the available variant-specific VE estimates from the 13 studies listed in Table 2. The single reported VE estimate from an RCT (Moderna COVE), whose symbol is differentiated by a dark outline, is the average (taken on the log scale and back-transformed to the VE scale) of the three variant-specific VE estimates in the Moderna COVE study. The solid black line is the y = x line. The Andrews et al. [11,12] TND estimates, which did not specify Omicron sublineage, were plotted against SVE results for Omicron BA.1, based on available genomic epidemiology data (see the Supplementary Material). BNT162b2 vaccine efficacy/effectiveness estimates were rescaled based on the difference from mRNA-1273 vaccine efficacy/effectiveness estimates (see details in Methods). For simplicity in plotting, both mRNA doses in the “any mRNA” regimens in Skowronski et al. [19] were assumed to be mRNA-1273, and no rescaling was performed. LLOD, lower limit of detection; mRNA, messenger RNA; PsV-nAB, pseudovirus neutralizing antibody; RCT, randomized, placebo-controlled efficacy trial; SVE, stochastic interventional vaccine efficacy; TND, test-negative design; VE, vaccine efficacy/effectiveness.

The SVE method was applied to the final randomized, double-blinded phase data of COVE, which followed participants for COVID-19 up to 126 days post dose 2. Fig. 2a shows SVE results, based on $\text{lo}{\text{g}}_{10}$ GMT ratio shifts δ from the observed D614G nAb ID50 titer value (GMT = 255 IU50/ml) and ranging from GMT = 14 IU50/ml to 2550 IU50/ml (D614G PsV-nAb ID50 measurements from 1041 vaccine recipients were included). Note that the labeling of points at GMT values of variants in Fig. 2a played no role in this data analysis; that labeling is only used for the next two sections that apply SVE analysis for predicting VE against variants. The result at δ = 0 corresponds to no shift, re-capitulating the direct empirical analysis of overall vaccine efficacy that does not account for nAb ID50 titer (VE = 92.9% [95% CI: 91.7, 93.9%]). For 1.6-fold, four-fold, and 10-fold shifts upwards of GMT, VE = 94.8% (95% CI: 93.8, 95.6%), 97.5% (95% CI: 96.7, 98.1%), and 99.3% (95% CI: 99.1, 99.4%), respectively. For 1.6-fold, four-fold, and 10-fold shifts downwards of GMT, VE = 88.2% (95% CI: 85.5, 90.4%), 84.7% (95% CI: 79.8, 88.4%), and 79.4% (95% CI: 70.1, 85.8%), respectively. VE significantly changed with shift $\text{δ}\left(\text{P }<\;0.001\right)$, supporting D614G nAb ID50 titer as a correlate of protection.

### Applying stochastic interventional vaccine efficacy analysis to modeling neutralizing antibody titer-predicted 2-dose vaccine efficacy against SARS-CoV-2 variants

Fig. 2a can be applied to predict two-dose VE against variant-specific COVID-19 for each of the nine (non-ancestral) variants characterized against two-dose vaccine recipient sera studied in Table 1. This is achieved by locating the GMT of two-dose vaccine-recipient sera against a given variant on the x-axis and taking the SVE estimate on the y-axis as the predicted VE against that variant. Each of the nine SVE estimates can be interpreted as the estimated vaccine efficacy had that specific variant been the only lineage circulating in the COVE trial and the background/placebo-arm risk of this variant were the same as that for ancestral observed in COVE. VE estimates against variants ranged from 67% (mu) to 86% (lambda) and were all lower than the estimate (93%) against the ancestral strain. VE estimates against kappa and beta were between 73% and 75%, while those against iota, gamma, delta, epsilon, and alpha, ranged between 82% and 84%.

### Exploratory analysis applying stochastic interventional vaccine efficacy analysis to modeling neutralizing antibody titer-predicted three-dose vaccine efficacy against SARS-CoV-2 variants

**Fig. 3.**
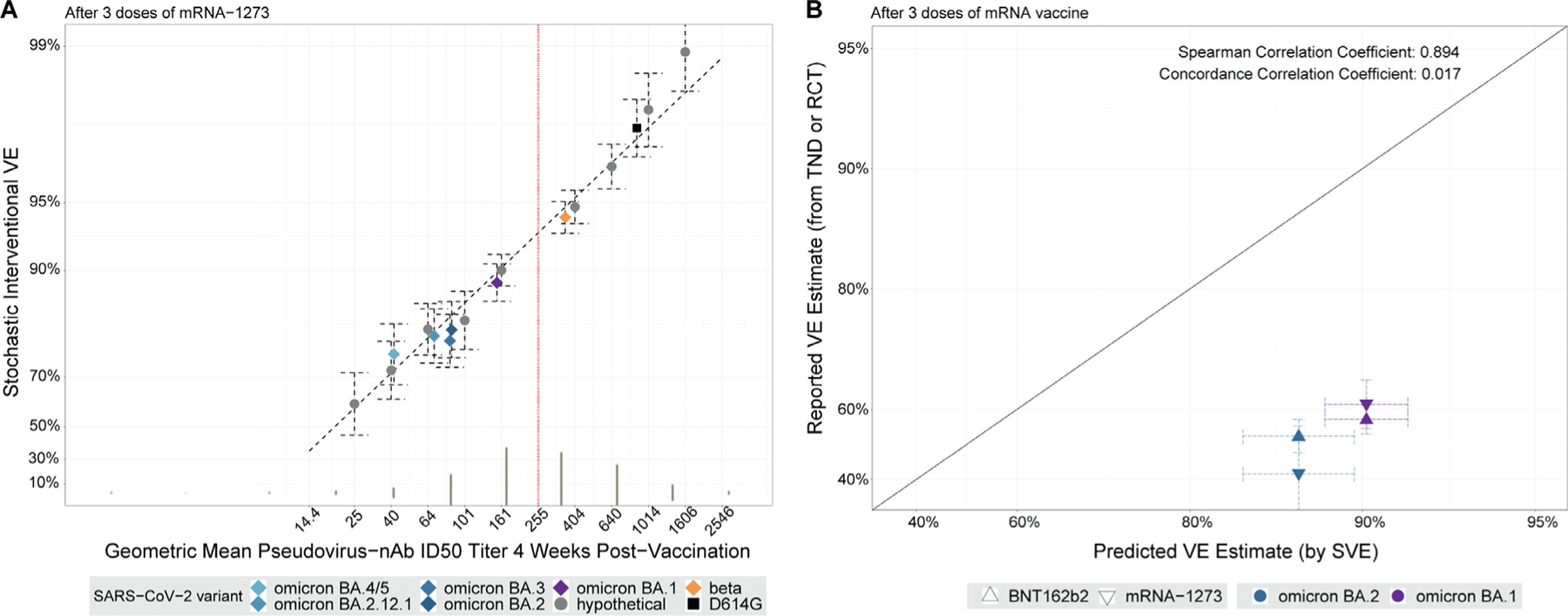
For three-dose mRNA-1273 recipients, stochastic interventional vaccine efficacy modeling of neutralization titer-predicted vaccine efficacy against different SARS-CoV-2 variants and validation analysis from external studies of three vaccine doses. (a) The y-axis plots the estimated vaccine efficacy against virologically confirmed, symptomatic COVID-19 disease for a vaccine that elicits geometric mean D29 post dose 3 PsV-nAb ID50 titer against the ancestral strain with the value indicated on the x-axis, estimated using the method in Hejazi et al. [1]. ID50 titer = IU/ml because ancestral strain titers were calibrated to the World Health Organization International Standard 20/136 D614G strain for anti-SARS-CoV-2 immunoglobulin. The black dots are the SVE estimates, with 95% confidence intervals (dashed lines), for a vaccine that hypothetically shifts each vaccine recipient’s $\text{lo}{\text{g}}_{10}$ nAb ID50 titer by a constant amount from their observed value against D614G that yields the given geometric mean D57 PsV-nAb ID50 titer against the designated variant (see Methods). For the colored diamonds, available phase I data (Table 1) are used to estimate the actual mean shift in geometric mean PsV-nAb ID50 titer for a response against D614G as opposed to against the designated SARS-CoV-2 variant. SVE estimates are reported for the follow-up period 7 through 100 days post day 57. The vertical green bars indicate the observed distribution of PsV-nAb ID50 titer as measured from participants post dose 2 in the COVE trial. The diagonal dashed line reports a linear model summary of how SVE(δ) is expected to change with $\text{lo}{\text{g}}_{10}$ geometric mean titer shifts (δ) (see the Supplementary Text for further details). (b) Comparison of VE estimates against SARS-CoV-2 infection or symptomatic COVID-19 caused by different SARS-CoV-2 variants obtained in TND studies of index-strain COVID-19 mRNA vaccines compared to the SVE estimates in panel A. Estimates are for after three doses of mRNA vaccine (mRNA-1273 and/or BNT162b2) vs placebo. The reported VE estimate for each plotted symbol corresponds to the average (taken on the log scale and back-transformed to the VE scale) of all three-dose variant-specific VE estimates from the three studies listed in Table 2 that reported these estimates. The solid black line is the y = x line. The Andrews et al. [11,12] TND estimates, which did not specify Omicron sublineage, were plotted against the SVE results against Omicron BA.1, based on the available genomic epidemiology data for SARS-CoV-2 variants (see Supplementary Material). BNT162b2 vaccine efficacy/effectiveness estimates were rescaled based on the difference from mRNA-1273 vaccine efficacy/effectiveness estimates (see details in Methods). For simplicity in plotting, the heterologous three-dose regimens were treated as three-dose mRNA-1273 regimens, with no rescaling performed. LLOD, lower limit of detection; mRNA, messenger RNA; PsV-nAB, pseudovirus neutralizing antibody; SVE, stochastic interventional vaccine efficacy; TND, test-negative design; VE, vaccine efficacy/effectiveness.

We next applied SVE to predict three-dose VE against variants. In Fig. 3a, the SVE estimate at δ = 0 is 97.3% (96.4, 97.9%), which can be interpreted as the vaccine efficacy that would have occurred in COVE against the ancestral strain that was circulating had the study tested three doses of the mRNA-1273 vaccine instead of two doses. For 1.2-fold, 1.8-fold, and three-fold shifts upwards of post-dose-3 GMT, VE = 97.5% (95% CI: 96.7, 98.1%), 98.2% (95% CI: 97.6, 98.6%), and 99.3% (95% CI: 99.1, 99.4%), respectively. For 1.4-fold, 2.2-fold, and 5.5-fold shifts downwards of post-dose-3 GMT, VE = 96.4% (95% CI: 95.5, 97.1%), 94.8% (95% CI: 93.8, 95.6%), and 90.7% (95% CI: 89.0, 92.1%), respectively.

An application of Fig. 3a is predicting three-dose VE against variant-specific COVID-19 for each of the seven variants characterized against three-dose vaccine recipient sera studied in Table 1. Each of the seven SVE estimates can be interpreted as the estimated VE had (1) that specific variant been the only lineage circulating in the COVE trial; (2) the background/placebo-arm risk of this variant been the same as for that observed for ancestral in COVE; and (3) the blinded period of the COVE trial studied three doses of vaccine vs three doses of placebo. The three-dose VE estimates against variants were all lower than the hypothetical three-dose VE estimate (97.3%) against ancestral. The lowest VE estimate (82%) was against omicron BA.4/BA.5. For other omicron variants, the VE estimates ranged from 90% to 85% for BA.1, BA.3, BA.2, and BA.2.12.1 in decreasing order. While these VE estimates may appear to reflect the stronger degree of protection conferred by three doses, this analysis is exploratory, requiring strong comparability (i.e., *ceteris paribus*) assumptions operationalized under the hypothetical of COVE studying a three-dose regimen during the blinded phase rather than the actual two-dose regimen and an analogous variant-invariant CoP model.

### Evaluation of prediction accuracy using vaccine efficacy/effectiveness estimates against variants

We next performed a validation analysis using empirical vaccine efficacy/effectiveness estimates (vs unvaccinated) against variants from external studies (Table 2). For two-dose vaccination (Fig. 2b), the Spearman rank correlation between predicted and empirical VE estimates was 0.143. The predictions tend to be underestimates, with low concordance: Concordance Correlation Coefficient (CCC) equal to 0.062. For three-dose vaccination (Fig. 3b), the Spearman rank correlation was 0.894; while this value is high, concordance was weak, and CCC was 0.017. The predictions tend to be overestimates, with predicted VEs ranging from 82–90% and empirical VE estimates from 40–60%.

### Reproducibility statement

All aspects of the statistical analysis were conducted using the open-source R language and environment for statistical computing and graphics (version 4.0.4). Code for replicating the analysis will be posted publicly on GitHub.

## Discussion

Our first objective was to evaluate post-dose 2 pseudovirus neutralizing antibody titer (PsV-nAb ID50) against D614G as a correlate of protection for the COVID-19 primary endpoint in the COVE study via the SVE approach. SVE sharply increased under stochastic shifts of geometric mean PsV-nAb ID50 titer upwards, and sharply decreased under downward shifts, further supporting the PsV-nAb ID50 biomarker as a correlate of protection [2]. The SVE approach has the advantage that it is readily possible to define meaningful potential population shifts of an immune marker distribution, because clinical studies of vaccine recipients can be used to define shifts that can be potentially produced by modifications of the vaccine regimen. Moreover, while our data analysis only considered shifts applied uniformly to all study participants, these shifts can depend on participant factors, such that the framework can accommodate situations where some shifts are possible for some sub-populations but not others. For example, higher immune marker levels may be attainable for younger vs older individuals, or for individuals seropositive vs seronegative to the pathogen under study. Given a division of the cohort into two subgroups, separate specified shifts ${\text{δ}}_{1}$ and ${\text{δ}}_{2}$ can be assigned to the subgroups, and a similar statistical analysis applied to estimate SVE(${\text{δ}}_{1}$, ${\text{δ}}_{2}$), which is vaccine efficacy under the ${\text{δ}}_{1}$ perturbation assigned subgroup 1 and the ${\text{δ}}_{2}$ perturbation assigned subgroup 2.

While our analysis focused on a COVID-19 VE trial, the SVE framework is equally relevant and applicable to an arbitrary VE trial for a given vaccine regimen and pathogen. However, details of the vaccine regimen, pathogen, immunoassay, and most importantly the biomarker derived from the immunoassay provide critical context for specifying the relevant marker shifts or shift functions for addressing particular objectives. For example, consider one biomarker with very broad dynamic range across vaccine recipients with quantitative values for all vaccine recipients, compared to another biomarker for which 50% of vaccine recipients have marker value before the assay lower limit of detection (LOD). For the latter biomarker, applying SVE analysis for a grid of constant shifts ranging from a decrease to the 5^th^ percentile to an increase to the 95^th^ percentile may be of interest, whereas for the latter biomarker, this analysis would not be useful and a more relevant approach would specify separate shift functions to the two subgroups with marker value below vs above the LOD. For another example, for a pathogen with limited diversity, the marker typically is a response to the vaccine antigen, where if the pathogen has high diversity, then the marker may be a cross-reactivity score to a panel of antigens.

Our second, totally distinct, objective evaluated how well SVE estimates from COVE could predict 2-dose mRNA VE against COVID-19 caused by different SARS-CoV-2 variants in the hypothetical context that a given variant had circulated in COVE. The SVE estimates were weakly concordant with empirical two-dose variant-specific VE estimates from external studies and tended to underestimate empirical VE. The relative lack of randomized, placebo-controlled trials (RCTs) providing empirical estimates of VE for this validation analysis partly explains the underestimation, with all but one of the studies being an observational test-negative design (TND), which, compared to RCTs, have been found to produce biased-upward estimates of VE against COVID-19 [24,26]. The ability of TND studies to unbiasedly estimate VE requires assumptions including that vaccination does not impact risk of other infectious diseases resulting in similar symptomatic illness, that individuals meeting the COVID-19 endpoint symptom criteria would not be more or less likely to seek COVID-19 testing if they were vaccinated, and that there is correct adjustment for confounding factors occurring in the absence of randomization to vaccine vs placebo. Especially for the three vaccine-dose TND studies [11,14,20], the comparator group of unvaccinated persons typically made up a small and highly selected proportion of the relevant population for inference. Moreover, the 95% CIs for vaccine effectiveness estimates from the TND studies are generally too narrow, reflecting uncertainty due to sampling variability but not accounting for other sources of uncertainty [27]. Other reasons for weak predictions are discussed in the Supplementary Material.

Our evaluation of how well SVE estimates could predict three-dose VE against omicron COVID-19 yielded overestimates. This over-estimation cannot be explained by the lack of RCTs. One potential explanation is that omicron may have a greater average challenge dose and/or greater viral infectivity (discussed in the Supplementary Material). However, we emphasize that the predictions of three-dose VE are only exploratory and make additional extrapolation assumptions beyond those made for the two-dose VE predictions.

Cromer et al. [26] found that a neutralization-CoP modeling approach performed well in predicting empirical vaccine effectiveness/VE against symptomatic COVID-19 caused by variants in TND, case-control, and RCT studies. Their modeled VE estimates had high correlation with empirical VE, with slight underestimation against non-omicron variants and overestimation against omicron beyond 3 months post-vaccination, consistent with our results. While both approaches are based on fold-change in geometric mean neutralization titer of vaccine recipient sera against a variant vs the index strain, our approach is based on one phase III study and one neutralization assay, while Cromer et al. [26]’s is based on different neutralization assays and used the geometric mean of convalescent sera to standardize readouts across studies. Moreover, we used nonparametric modeling of individual-level data while Cromer et al. used parametric modeling of study-level data. Additional differences are detailed in the Supplementary Material. While our approach is theoretically statistically optimal for robustness and efficiency [1], the limited sample size of the omicron validation studies hinders performance comparison.

We draw two conclusions for the second objective, the first being the above-noted evidence against the postulated “variant-invariant CoP model.” Based on the low concordance of SVE estimates with empirical variant-specific VE estimates, our second conclusion is that the prediction modeling ventured a “bridge too far,” caused in part by the hypothetical context for the predictions, the limited accuracy and precision of variant-specific VE estimates in the TND observational studies as noted above, and variations among the TND studies. Another reason may be that our study did not directly assess a ‘variant-matched correlate’ that would associate variant-specific titer with incidence of variant-matched COVID-19, as nAb assay data against D614G may have limited ability to predict efficacy against other variants. This hypothesis will be tested in future work. In contrast, it was much easier to attain success for our first objective, given that it is based wholly on data directly observed in COVE with fewer assumptions and avoiding extrapolations. We conclude that the SVE framework is a promising new approach for predicting how VE would change under specified perturbations of an immune marker’s distribution, where prediction domain application details may determine success. One potential future application is modeling predicted improvement in VE with updated vaccine-insert sequence(s).

## Supplementary Material

MMC1

## Figures and Tables

**Table 1:** Studies from which serum samples were obtained for nAb assays, variants assayed, and sample sizes.

Study with serum samples	Trial phase	Vaccine (no. doses)	Sampling timepoint for assay	Spike variant/subvariant used in the nAb assay^a^	No. participants with nAb ID50 data
El Sahly et al. [7]	Phase III	mRNA-1273 (two)	4 weeks post second dose	Ancestral (D614G) Beta^b^ Omicron (BA.1)	30 30 30
Atmar et al. [28]	Phase I/II	mRNA-1273 (three)	2 weeks post third dose	D614G Beta Delta Omicron (BA.1)	10 10 10 10
DMID21–0012 Lyke et al. [29]	Phase I/II	mRNA-1273 (three)	4 weeks post third dose	D614G Omicron (BA.1) Omicron (BA.2) Omicron (BA.3) Omicron (BA.2.12.1) Omicron (BA.4/BA.5)	16 16 16 16 16 16
Anderson et al. [30]	Phase I	mRNA-1273 (two)	4 weeks post second dose	D614G Alpha^c^ Alpha/E484K^c^ Beta Delta_AY.1^d^ Delta_AY.2^d^ Delta_AY.3^d^ Epsilon Gamma Iota Kappa Lambda Mu	28 28 10 26 10 10 28 28 25 28 28 10 10

mRNA, messenger RNA; nAb, neutralizing antibody

a Supplementary Table 1 provides a list of mutations relative to the index virus (NCBI Ref: NC_045512.2) for each variant.

b The Beta version used for testing phase III samples contains 246R in Spike, whereas the Beta used to test phase I samples contains an R246I mutation. No difference was found in neutralizing susceptibility between the two versions of Beta (246R vs 246I) (Montefiori lab, unpublished).

c Data were combined to determine a single geometric mean titer shift for alpha in Fig. 2a.

d Data were combined to determine a single $\text{lo}{\text{g}}_{10}$ geometric mean titer shift value of delta in Fig. 2a.

**Table 2:** Empirical variant-specific vaccine efficacy/vaccine effectiveness estimates obtained from test-negative design observational studies and from one randomized controlled trial used in the validation analysis^∗^

Reference	Design	Setting	Vaccine, doses	Endpoint/Outcome	Follow-up period post last dose	Relevant calendar dates for data used	Variant	Vaccine effectiveness or Vaccine efficacy (vs. unvaccinated)
Pajon et al. [8]	RCT	U.S.	mRNA-1273 (two)	Symptomatic disease^A^	35 to 126 days	Data on COVID-19 cases from Jul 27, 2020 to Mar 26, 2021	Epsilon (B.1.427 and B.1.429)	81.2 (36.1, 94.5)^B^
Lopez Bernal et al. [17]	TND	England; national healthcare registries	BNT162b2 (two)^∗∗^	Symptomatic disease^C^	14 to 140 days^D^	Data on COVID-19 vaccinations through May 30, 2021; Data on SARS-CoV-2 PCR tests from Oct 26, 2020 to May 30, 2021	Alpha	93.7 (91.6, 95.3)^E^
							Delta	88 (85.3, 90.1)^E^
Bruxvoort et al. [13]	TND	Kaiser Permanente Southern CA; integrated healthcare system	mRNA-1273 (two)	SARS-CoV-2 infection^F^	45 days to ∼6.5 months^G^	Data on SARS-CoV-2 PCR tests from Mar 1, 2021 to Jul 27, 2021	Alpha	98.4 (96.9, 99.1)^I^
					14 to 120 days^H^		Delta	90.2% (86.1%, 93.1%)^H^
					45 days to ∼6.5 months^G^		Epsilon	97.6 (90.2, 99.4)^I^
					45 days to ∼6.5 months^G^		Gamma	95.5 (90.9, 97.8)^I^
					45 days to ∼6.5 months^G^		Iota	95.7 (81.7, 99.0)^I^
					45 days to ∼6.5 months^G^		Mu	90.4 (73.9, 96.5)^I^
Abu-Raddad et al. [10]	TND	Qatar; national health databases	BNT162b2 (two)	SARS-CoV-2 infection^J^	14 to 66 days^K^	Data on COVID-19 testing, vaccination, and clinical infection from Feb 1, 2021 to Mar 31, 2021	Alpha	89.5 (85.9, 92.3)^L^
						As above, except SARS-CoV-2 sequencing data for beta only available for Mar 8, 2021 to Mar 31, 2021	Beta	75.0 (70.5, 78.9)^L^
Andrews et al. [11]	TND	England; national healthcare registries	BNT162b2 (two)^∗∗^	Symptomatic disease^M^	5 to 19 weeks^N^	Data on people who reported symptoms and underwent SARS-CoV-2 PCR testing from Nov 27, 2021 to Jan 12, 2022	Delta	80.1 (79.2, 80.9)^N^
			BNT162b2 (three)		5 to ≥ 10 weeks^O^		Omicron (BA.1/BA.2)	32.8 (31.3, 34.2)^N^
							Delta	90.7 (90.4, 91.3)^O^
			mRNA-1273 (two)		5 to 19 weeks^N^		Omicron (BA.1/BA.2)	50.6 (49.7, 51.5)^O^
							Delta	86.9 (85.2, 88.5)^N^
			mRNA-1273 (three)		2–4 weeks		Omicron (BA.1/BA.2)	39.0 (35.5, 42.3)^N^
							Delta	96.4 (91.4, 98.5)^P^
			BNT162b2 (two)/mRNA-1273 (one)		5–9 weeks		Omicron (BA.1/BA.2)	66.3 (63.7, 68.8)^P^
							Delta	94.9 (93.0, 96.2)^Q^
			mRNA-1273 (two)/BNT162b2 (one)		2–4 weeks		Omicron (BA.1/BA.2)	64.4 (62.6, 66.1)^Q^
							Delta	94.7 (89.3, 97.3)^R^
							Omicron (BA.1/BA.2)	64.9 (62.3, 67.3)^R^
Nasreen et al. [18]	TND	Canada; community-dwelling individuals. Province-wide (Ontario) datasets for public health surveillance, vaccination, and healthcare system use	BNT162b2 (two)^∗∗^	Symptomatic infection^S^	7 days to 7 months^T^	Data on SARS-CoV-2 test results in symptomatic individuals Dec 14, 2020 to Aug 3, 2021	Alpha	89 (87, 90)^X^
					7 days to 7 months^T^		Beta	87 (8, 98)^X^
					7 days to 7 months^T^		Gamma	88 (73, 94)^X^
					91 days to 7 months^U^		Delta	92 (90, 94)^X^
			mRNA-1273 (two)		7 days to ∼6.5 months^V^		Alpha	92 (88, 95)^X^
					7 days to ∼6.5 months^V^		Beta	100 (NA, 100)^X^
					7 days to ∼6.5 months^V^		Gamma	100 (NA, 100)^X^
					70 days to ∼6 months^W^		Delta	95 (91, 97)^X^
Chemaitelly et al. [16]	TND	Qatar; national health databases	mRNA-1273 (two)	SARS-CoV-2 infection^Y^	14 to 105 days^Z^	Data on COVID-19 cases by variant type Alpha for Feb 1, 2021 to May 10, 2021	Alpha	100.0 (91.8, 100.0)^BB^
					42 to 105 days^AA^	Data on COVID-19 cases by variant type Beta for Mar 8, 2021 to May 10, 2021	Beta	96.4 (91.9, 98.7)^BB^
Andrews et al. [12]	TND	England; national healthcare registries	BNT162b2 (two)	Symptomatic disease^CC^	14 days to ∼ 6 months^DD^	Data on positive PCR tests from Dec 8, 2020 to Oct 1, 2021.	Alpha	94.9 (91.4, 96.9)^DD^
					2 to 19 weeks^EE^		Delta	82.4 (82.1, 82.6)^EE^
			mRNA-1273 (two)		2 to 14 wks^FF^		Delta	90.6 (89.7, 91.4)^FF^
Chemaitelly et al. [15]	TND	Qatar; national health databases	BNT162b2 (two)	SARS-CoV-2 infection^GG^	30 to 150 days^HH^	Data on positive PCR tests from Jan 1, 2021 to Sept 5, 2021	Alpha	73.6 (50.7, 85.6)^HH^
							Beta	60.0 (55.5, 76.0)^HH^
							Delta	63.6 (49.7, 73.6)^HH^
Chemaitelly et al. [14]	TND	Qatar; national health databases	BNT162b2 (two)	Symptomatic infection^II^	1 to 3 months^JJ^	Dec 23, 2021 to Feb 28, 2022	Omicron (BA.1)	46.6 (33.4, 57.2)^JJ^
							Omicron (BA.2)	51.7 (43.2, 58.9)^JJ^
			BNT162b2 (three)		∼1 to 2 months^KK^		Omicron (BA.1)	40.5 (30.8, 48.8)^KK^
							Omicron (BA.2)	40.2 (34.2, 45.7) ^KK^
			mRNA-1273 (two)		1 to 3 months^JJ^		Omicron (BA.1)	71.0 (24.0, 89.0)^JJ^
							Omicron (BA.2)	35.9 (−5.9, 61.2)^JJ^
			mRNA-1273 (three)		∼1 to 1.5 months^LL^		Omicron (BA.1)	45.3 (17.8, 63.5)^LL^
							Omicron (BA.2)	41.9 (23.4, 56.0)^LL^
Tseng et al. [20]	TND	Kaiser Permanente Southern CA; integrated healthcare system	mRNA-1273 (two)	SARS-CoV-2 infection^MM^	14 to 180 days^NN^	Dec 6, 2021 to Dec 31, 2021	Delta	69.9 (64.7, 75.6)^NN^
					14 to 180 days		Omicron (BA.1)	35.0 (25.0, 41.9)^NN^
				mRNA-1273 (three)	14 to *>*60 days^OO^		Delta	90.7 (86.9, 93.2)^OO^
				SARS-CoV-2 infection^PP^	14 to *>*60 days^OO^		Omicron (BA.1)	61.4 (57.6, 65.0)^OO^
Tartof et al. [25]	TND	Kaiser Permanente Southern CA; integrated healthcare system	BNT162b2 (two)		37 to 126 days^QQ^	Dec 14, 2020 to Aug 8, 2021	Delta	78.1 (70.0, 83.8)^QQ^
Skowronski et al. [19]	TND	British Columbia, Canada; community-based assessment centers, ER rooms, hospitals, other sites	mRNA-1273 and/or BNT162b2 (any mRNA vaccine) (two)^RR^	SARS-CoV-2 infection^SS^	28 to 139 days^TT^	May 30, 2021 to Nov 27, 2021	Delta	90.0 (89.4, 90.0)^TT^
		Quebec, Canada; community-based assessment centers, ER rooms, hospitals, other sites	mRNA-1273 and/or BNT162b2 (any mRNA vaccine) (two)^RR^	SARS-CoV-2 infection^SS^	28 to 139 days^TT^	May 30, 2021 to Nov 27, 2021	Delta	88.1 (87.4, 88.7)^TT^

TND = test-negative design; RCT = randomized controlled trial.

∗ Only variant-specific vaccine efficacy/vaccine effectiveness estimates meeting the eligibility criteria for inclusion in the validation analysis (detailed in the supplementary material) are listed here.

∗∗ Potential significant heterogeneity in the interval between first and second dose due to e.g. adoption of delayed second-dose strategy. The dose interval could extend up to 12 weeks in some cases.

A Laboratory-confirmed SARS-CoV-2 infection detected by real-time reverse transcription polymerase chain reaction.

B From Table 2 in Pajon et al.

C PCR-confirmed SARS-CoV-2 infection with symptoms consistent with COVID-19 (high temperature, new continuous cough, or loss or change in sense of smell or taste).

D See Figure S3 in Lopez Bernal et al.; all COVID-19 endpoints were between 14 days to 140 days post dose 2.

E Overall reported vaccine effectiveness estimates from Table 2 in Lopez Bernal et al.

F With or without symptoms; positive SARS-CoV-2 RT-PCR test.

G mRNA-1273 was first made available on Dec 18, 2020. With second doses on approximately Jan 15, 2021 and data on tests from Mar 1, 2021 through Jul 27, 2021, follow up period post dose 2 extended from 45 days through approximately 6.5 months.

H For the delta variant only, vaccine effectiveness of 2 doses of mRNA-1273 was reported for the time intervals 14–60 days, 61–90 days, 91–120 days post dose 2 (Table S12 in Bruxvoort et al.). The average of the VE point estimates and the average of the 95% confidence limits [averaged on the log(1-VE) scale] over the three time intervals is reported as the vaccine effectiveness estimate against delta (right hand column) of this table..

I Estimates from Figure 1 in Bruxvoort et al.

J Positive SARS-CoV-2 PCR test.

K Vaccinations started Dec 20, and endpoint ascertainment finished March 18. With second doses around Jan 11 (Dec 20 + 21 days), vaccine effectiveness was studied between 14 through 66 days post dose 2.

L Estimates from Table 1 in Abu-Raddad et al.

M Self-reported symptoms consistent with COVID-19 (high temperature, new continuous cough, or loss or change in sense of smell or taste) with positive SARS-CoV-2 PCR test.

N Vaccine effectiveness of 2 doses of BNT162b2 and mRNA-1273 was reported for the time intervals 5–9, 10–14, and 15–19 weeks post dose 2 (Table 3 in Andrews et al.). The average of the VE point estimates and the average of the 95% confidence limits [averaged on the log(1-VE) scale] over the three time intervals is reported as the vaccine effectiveness in this table (right hand column).

O Vaccine effectiveness of 3 doses of BNT162b2 was reported for the time intervals 5–9 and ≥ 10 weeks post dose 2 (Table 3 in Andrews et al.). The average of the VE point estimates and the average of the 95% confidence limits [averaged on the log(1-VE) scale] over the two time intervals is reported as the vaccine effectiveness in this table (right hand column).

P Vaccine effectiveness of 3 doses of mRNA-1273 was reported for the time interval 2–4 weeks post boost (Table 3 in Andrews et al.).

Q Vaccine effectiveness of 2 doses of BNT162b2 followed by an mRNA-1273 boost was reported for the time interval 5–9 weeks post boost (Table 3 in Andrews et al.).

R Vaccine effectiveness of 2 doses of mRNA-1273 followed by a BNT162b2 boost was reported for the time interval 2–4 weeks post boost (Table 3 in Andrews et al.).

S Laboratory-confirmed SARS-CoV-2 infection detected by real-time reverse transcription polymerase chain reaction.

T BNTb162b became available on Dec 14, 2020. With second doses around Jan 4, 2022, follow-up time post dose 2 (through Aug 3, 2021) was approximately 7 months.

U BNTb162b became available on Dec 14, 2021. For delta, test-positive cases and test-negative controls were restricted to those tested on/after the dates of initial confirmation of the delta variant in Ontario, which was April 5, 2021. With second doses around Jan 4, 2021, follow-up time post dose two was thus 91 days to ∼6.5 months.

V mRNA-1273 became available on Dec 28, 2020. With second doses around Jan 25, 2021, follow-up time post dose 2 (through Aug 3, 2021) was approximately 6.5 months.

W mRNA-1273 became available on Dec 28, 2020. For delta, test-positive cases and test-negative controls were restricted to those tested on/after the dates of initial confirmation of the delta variant in Ontario, which was April 5, 2021. With second doses around Jan 25, 2021, follow-up time post dose 2 (through Aug 3, 2021) was approximately 6 months.

X Vaccine effectiveness estimates from Figure 1 in Nasreen et al. are reported.

Y PCR-positive swab regardless of the reason for PCR testing or presence of symptoms.

Z mRNA-1273 was first available on Dec 28, 2020. Adding 28 days, this corresponds to dose 2 on approximately Jan 25, 2021. Cases (alpha variant) were recorded through May 10, 2021, giving a follow-up period post dose 2 of 14 to 105 days.

AA For beta the same, except data on beta cases started from Mar 8, 2021 (=42 days post dose 2), yielding a follow-up period post dose 2 of 14 to 105 days.

BB Vaccine effectiveness estimates from Table 3 in Chemaitelly et al.

CC Self-reported symptoms consistent with COVID-19 (high temperature, new continuous cough, or loss or change in sense of smell or taste) with positive SARS-CoV-2 PCR test.

DD Vaccine effectiveness against alpha of 2 doses of BNT162b2 (16+ y) was reported for the time intervals 2–9 and 10+ weeks post dose 2 (Table S11 in Andrews et al.). The average of the VE point estimates and the average of the 95% confidence limits [averaged on the log(1-VE) scale] over the two time intervals is reported as the vaccine effectiveness in this table (right hand column). Vaccines were first available early Dec with a 3-week interval at first, meaning dose 2 was on approximately Dec 22, 2021. Table S2 of Andrews et al. provides the period for the cases/controls included in the analysis set for VE against alpha symptomatic infection as Jan 4, 2021 through Jun 27, 2021, meaning the follow-up post dose 2 extends to approximately 6 months.

EE Vaccine effectiveness against delta of 2 doses of BNT162b2 (16+ y) was reported for the time intervals 2–9, 10–14, and 15–19 weeks post dose 2 (Table 1 in Andrews et al.). The average of the VE point estimates and the average of the 95% confidence limits [averaged on the log(1-VE) scale] over the three time intervals is reported as the vaccine effectiveness in this table (right hand column).

FF Vaccine effectiveness against alpha of 2 doses of mRNA-1273 (16+ y) was reported for the time intervals 2–9 and 10–14 weeks post dose 2 (Table S10 in Andrews et al.). The average of the VE point estimates and the average of the 95% confidence limits [averaged on the log(1-VE) scale] over the two time intervals is reported as the vaccine effectiveness in this table (right hand column).

GG PCR-positive swab regardless of the reason for PCR testing or presence of symptoms.

HH Vaccine effectiveness against alpha of 2 doses of BNTb162b were reported for the time intervals 1^st^ month, 2^nd^ month, 3^rd^ month, 4^th^ month, and 5^th^ month post dose 2 (Table S5 in Chemaitelly et al.). The average of the VE point estimates and the average of the 95% confidence limits [averaged on the log(1-VE) scale] over the five time intervals is reported as the vaccine effectiveness in this table (right hand column).

II PCR-positive nasopharyngeal swab conducted because of clinical suspicion due to presence of symptoms compatible with a respiratory tract infection.

JJ Estimates for “1–3 months after Dose 2 and no Dose 3” for BTN162b2 and mRNA-1273 against BA.1 or BA.2 symptomatic infection were reported in Tables 3 and 4, respectively, of Chemaitelly et al.

KK Estimates for “≥1 month after Dose 3” for BTN162b2 against BA.1 or BA.2 symptomatic infection were reported in Table 3 of Chemaitelly et al. The median date of 3^rd^ dose receipt was Dec 27, 2021, yielding ∼1–2 months as the approximate follow up period post boost (Feb 28, 2022 was the end of the study).

LL Estimates for “≥1 month after Dose 3” for mRNA-1273 against BA.1 or BA.2 symptomatic infection were reported in Table 4 of Chemaitelly et al. The median date of 3^rd^ dose receipt was Jan 16, 2022, yielding ∼1–1.5 months as the approximate follow up period post boost (Feb 28, 2022 was the end of the study).

MM Requested SARS-CoV-2 testing for any reason, with or without symptoms, and tested positive by the RT–PCR TaqPath COVID-19 kit.

NN Vaccine effectiveness estimates for “14–90 days” and “91–180 days” post dose 2 were reported in Table 2 of Tseng et al. The average of the VE point estimates and the average of the 95% confidence limits [averaged on the log(1-VE) scale] over the two time intervals is reported as the vaccine effectiveness in this table (right hand column).

OO Vaccine effectiveness estimates for “14–60 days” and “*>*60 days” post dose 3 were reported in Table 2 of Tseng et al. The average of the VE point estimates and the average of the 95% confidence limits [averaged on the log(1-VE) scale] over the two time intervals is reported as the vaccine effectiveness in this table (right hand column).

PP Defined as testing positive for SARS-CoV-2 via a PCR test from any sample (ie, bronchial lavage, nasopharyngeal or nasal swab, oropharyngeal swab, throat swab, saliva, sputum, or tracheal aspirate) in any clinical setting regardless of the presence of symptoms.

QQ Vaccine effectiveness estimates for “37 to 66 days”, “67 to 96 days”, and “97 to 126 days” post dose 2 were reported in Appendix Table 8 of Tartof et al. The average of the VE point estimates and the average of the 95% confidence limits [averaged on the log(1-VE) scale] over the three time intervals is reported as the vaccine effectiveness in this table (right hand column).

RR The interval between the first and second dose varied throughout the study, as described in the Introduction of Skowronski et al.

SS Positive SARS-CoV-2 nucleic acid amplification test (NAAT).

TT Vaccine effectiveness estimates against Delta infection for each province for “28–55”, “56–83”, “84–111”, and “112–139” post dose 2 were reported in Supplementary Table 14 of Skowronski et al. The average of the VE point estimates and the average of the 95% confidence limits [averaged on the log(1-VE) scale] over the three time intervals is reported as the vaccine effectiveness in this table (right hand column).
