# Supplementary material for: Stochastic interventional approach to assessing immune correlates of protection: Application to the COVE messenger RNA-1273 vaccine trial
Source: Int J Infect Dis. Author manuscript; Available in PMC 2024 Feb 5. (PMC10841741; doi:10.1016/j.ijid.2023.09.012)
Supplement: MMC1 [file NIHMS1936691-supplement-MMC1.docx]

**Supplementary Material**

***Introduction***

*Evidence for neutralizing antibody (nAb) titer as a correlate of protection (CoP) for COVID-19 vaccines*

The body of evidence supporting nAb titer against the index or D614G strain as a CoP for vaccines against symptomatic COVID-19 includes: 1) natural history studies correlating infection-induced neutralizing antibodies (nAbs) against the index strain with protection from SARS-CoV-2 infection,^1^ 2) mechanistic studies in animal models consisting of nAb passive/adoptive transfer followed by SARS-CoV-2 challenge,^2,3^ 3) COVID-19 vaccine challenge studies in animal models correlating post-vaccination nAbs [where specified^2^ against D614G (defined as the index strain harboring the D614G mutation)] with protection from SARS-CoV-2 replication after challenge with the USA-WA1/2020 strain,^2,4^ 4) passive administration of monoclonal nAbs in humans demonstrating protection from symptomatic COVID-19,^5^ 5) correlates analyses of phase 3 COVID-19 vaccine efficacy trials quantifying the relationship between post-vaccination nAb levels (assessed against D614G) with protection from symptomatic COVID-19,^6-10^ and 6) meta-analyses of COVID-19 vaccine efficacy trials correlating mean post-vaccination nAb levels with population-level protection from symptomatic COVID-19.^11,12^

***Methods***

*Stochastic interventional vaccine efficacy (SVE) analysis*

For specific methodological details on the SVE approach, see the COVID-19 Prevention Network (CoVPN) immune correlates VE trial marker statistical analysis plan (SAP)^13^ and the statistical methods for estimating counterfactual risk.^14^

**Assumptions for estimation of post dose 2 VE:** The following standard assumptions are made in applying the SVE approach to estimate post dose 2 VE against the COVID-19 primary endpoint of the COVE study under a given mean shift in log_10_ PsV-nAb ID50 titer. For individuals in the vaccine arm (for whom A = 1), within all strata defined by the baseline adjustment variables X, it is necessary for the modified immune response S + δ to be observed – that is, observation of immune response S within the stratum X must imply observability of S + δ in stratum X – for SVE to be estimable. This first condition may be evaluated in practice because violations lead to notable instability in SVE estimates. Additionally, in the vaccine arm, the set of baseline adjustment variables X must be rich enough that there are no unmeasured confounders of the relationship between S and Y and no confounders of the relationship between S and Y that are affected by vaccination A. This second condition may be evaluated in practice without overly stringent assumptions by way of nonparametric sensitivity analyses (e.g., E-values).^15,16^

**Assumptions for prediction of post dose 3 VE:** We also consider prediction of post dose 3 VE (vs. 3 doses of placebo) against COVID-19 with a specific variant for which the hypothetical variant trial is modified to a new version of the COVE study that envisages both (1) 3 vaccine doses instead of 2 doses; and (2) the variant had been the sole circulating viral lineage during the trial (instead of the ancestral lineage) and that its post dose 3 placebo-arm incidence of variant-specific COVID-19 was the same as the post dose 2 placebo-arm incidence of D614G-virus COVID-19 in the actual COVE study (once again a *scenario* for consideration, not an assumption). The variant-invariant model for 3 vaccine doses is the same as for 2 vaccine doses, except the last phrase becomes “when assigning all participants to 3 doses of the mRNA-1273 vaccine (without a shift) in the 3-dose hypothetical variant trial.” The variant-invariant CoP model for 3 vaccine doses involves more extrapolation than the variant-invariant CoP model for 2 vaccine doses, such that predictions of 3-dose VE are less empirically based, less precise, and more susceptible to bias than the predictions of 2-dose VE. Therefore, we consider our 2-dose VE predictions as primary analyses and the 3-dose VE predictions as hypothesis-generating exploratory analyses.

**Marginal structural models:** As noted in the main text, a simple linear summary measure may be used to describe the relationship between VE estimates SVE(δ) and the corresponding geometric mean titer (GMT) shifts δ; this is displayed as the diagonal dashed line in panel A of Figure 2 (post dose 2 estimates) and Figure 3 (post dose 3 estimates). This linear summary measure is constructed by way of a nonparametric marginal structural model (MSM),^14,17,18^ which takes the form E[SVE(δ) | δ] = β0 + β1 δ, where E() is the expectation operator. In this simple MSM, the slope parameter β1 may be interpreted as the expected change in SVE(δ) for unit-level changes in the shift value δ. Recalling that for the main post dose 2 and exploratory post dose 3 SVE analyses we work with log10-transformed GMT of pseudovirus (PsV) neutralizing antibody (nAb) ID50, a shift value of δ = 1 corresponds to a hypothetical individual-level order of magnitude change in PsV nAb ID50 GMT. Consideration of a range of such δ shifts yields SVE-based counterfactual VE estimates, one for each hypothetical shift value δ (across a grid of δ); how this corresponding grid of SVE estimates changes for various shifts δ serves as a convenient summary measure of the impact that the PsV nAb ID50 correlate has on VE. The MSM slope parameter β1 can then be used to evaluate the sensitivity of VE to hypothetical or variant-induced changes (relative to D614G) in PsV nAb ID50. Building on this framing, a hypothesis test of this slope parameter β1, which evaluates evidence for the null hypothesis H0: β1 = 0 against the two-sided alternative H1: β1 ≠ 0, provides a formal way (complete with a p-value) to assess how VE may be expected to change for shifts in the immune marker. In principle, this hypothesis test can also be inverted to provide a corresponding Wald-style confidence interval for β1.

**Positivity requirements:** In order to estimate the δ-specific VE – that is, SVE(δ) – using the SVE approach described, it is necessary to compute the conditional density of the immune response S given baseline covariates X, q(S | A = 1, X), and the same conditional density under a corresponding shift δ, q(S – δ | A = 1, X); for technical details, we refer to refs.^14,19,20^ Saliently, the positivity condition for the SVE parameter can be cast in terms of a ratio of these conditional densities, that is, as q(S – δ| A = 1, X) / q(S | A = 1, X), which remains stable so long as the conditional density in the denominator is bounded away from zero. For practical reasons, we adopt an additional positivity condition in our SVE analysis. For a given shift value δ, we allow for the post-shift (counterfactual) immune response, S + δ, to fall below the LLOD for at most 10% of study units. As ought to be expected, this criterion impacts only negative shift values, δ< 0, and impacts those values for which the magnitude of δ is relatively large more, resulting in SVE(δ) estimates for relatively large, negative δ possibly being considered unstable. The rationale for this criterion is as follows: All values of δ for which the counterfactual S + δ < LLOD are essentially identical, as, by definition, distinguishing between immune response measurement values below the assay’s LLOD is impossible. So, in principle, SVE(δ) should take on only a single value for all values of δ for which S + δ < LLOD, and, when this condition holds for a significant proportion of units, SVE(δ) fails to convey any interesting information about how VE may change; moreover, it may suffer from certain identifiability assumptions failing to hold. Acknowledging that the choice of cut-off for which it may be deemed that a significant proportion of study units’ shifted immune response values fall below LLOD is arbitrary, we choose 10% for this proportion. Having made this choice, for our SVE-based analyses, only the shift value δ corresponding to PsV nAb GMT change (vs. D614G) for omicron (BA.1) post dose 2 violates this criterion and is thus dropped from the analytic results reported in Figure 2.

*Literature search for vaccine effectiveness/efficacy estimates for validation analysis*

To identify test negative design (TND) studies and randomized controlled vaccine efficacy trials with estimates of vaccine effectiveness or vaccine efficacy, respectively, against a single SARS-CoV-2 variant for inclusion in the validation analysis, PubMed was queried in June 2022 using various combinations of the search terms “COVID-19”, “efficacy”, “effectiveness”, “SARS-CoV-2”, “vaccine”, and “variant”. From the identified studies, as well as those included in a meta-analysis of test-negative design studies,^21^ the following criteria were applied to filter for eligible vaccine effectiveness or vaccine efficacy estimates for inclusion in the validation analysis:

- BNT162b2 or mRNA-1273 vaccine was administered, at 2 or 3 doses (both homologous and heterologous regimens were considered)
- SARS-CoV-2 sequencing was performed to identify the variant against which vaccine effectiveness/efficacy was estimated^1^ (S gene target failure was also accepted as a proxy for Omicron identification^2^)
- The RCT/TND-reported point estimate of variant-specific VE was against a single variant (i.e. not against 2 variants pooled)^3^
- Vaccine effectiveness/efficacy was assessed using an placebo or unvaccinated comparison group and studied efficacy against outcomes occurring from between 1 to ~7 months post dose 2 or post dose 3^4^
- The RCT/TND reported point estimate of variant-specific VE was based on a total of at least 15 endpoint cases across both the vaccine and placebo arms (for RCT) or at least 15 test-positive cases across both vaccinated and unvaccinated individuals (for TND)^5^

^1^ If SARS-CoV-2 sequencing was performed for only a portion of the study and then a given variant was assumed for the remainder of the study based on exceeding 99% of all sequenced cases, the study was included.

^2^ Based on the fact that the US FDA and WHO advised that S gene target failure from select COVID-19 RT–PCR assays can be used as a screening method for Omicron^22,23^ and that S gene target failure has served as a proxy in the United Kingdom for identifying Omicron^24,25^

^3^ The variant-specific estimate could include two lineages of the same variant, i.e. the VE estimate against epsilon (B.1.427 and B.1.429) was selected from the Pajon et al. study^26^ instead of the epsilon B.1.429 estimate, due to the increased number of total endpoint cases (18 vs. 12). However, estimates against two variants, e.g. gamma and epsilon, were not included.

^4^ If for 3-dose recipients, vaccine effectiveness estimates were only provided for an earlier time frame (e.g. 2-4 weeks post 3^rd^ dose), the estimates through the earlier time frame were used as noted in Table 2.

^5^ In the case of zero test-positive vaccinated cases and an unreported number of test-positive unvaccinated cases, the criterion of >= 15 cases across test-positive vaccinated and test-positive unvaccinated individuals was assumed to be met if there were >= 5000 test-negative vaccinated controls.

Due to variation in dosing intervals, e.g. delayed administration of dose 2 in some countries, flexibility was allowed in the dosing interval (from dose 1 to 2 in the 2-dose studies and from dose 2 to 3 in the 3-dose studies). Studies with substantial heterogeneity in this interval, which could extend to 12 weeks, are identified in Table 2.

If vaccine effectiveness estimates were given that had been adjusted for covariates (in addition to unadjusted estimates), the adjusted estimates were chosen.

*Genomic epidemiology data*

In Figures 2, 3: Information on SARS-CoV-2 variants that were circulating in the United Kingdom during the study period (Nov. 27, 2021 to Jan. 12, 2022) was obtained using Nextstrain (GISAID data).^27^

***Discussion***

*Use of the uniform shift function*

We considered a grid of geometric mean shifts of the entire study population consistent with the observed data on neutralization titer against D614G and did not modify the shift depending on baseline factors. Use of a uniform shift function is partly justified by the fact that the COVE study population enrolled few immunocompromised participants and similar neutralizing antibody titers were elicited in mRNA-1273 recipients aged ≥ 71 years compared to those aged 18-55 years.^28^

*Potential reasons for weak prediction of 2-dose VE against variants*

The 95% confidence intervals for vaccine effectiveness estimates from the TND studies are generally too narrow, reflecting only uncertainty due to sampling variability but not accounting for other sources of uncertainty.^29^ Other reasons for the weak predictions of how well SVE estimates from COVE could predict 2-dose mRNA VE against COVID-19 caused by different SARS-CoV-2 variants (in the hypothetical context that a given variant had circulated in COVE) include different study populations and different study endpoints in COVE compared to the validation studies. For example, in COVE, essentially all participants included in the analysis were naïve to SARS-CoV-2, whereas in the vaccine effectiveness studies some participants were previously infected. Therefore, the fact that vaccine effectiveness is generally higher in previously infected than naïve populations^30,31^ partly explains the under-predictions. Additionally, our predictions are made under a variant-invariant CoP model, and lack of fit of this model (e.g., due to heterogeneity in viral infectivity across variants) would lead to imperfect predictions. Moreover, the PsV-nAb ID50 biomarker is an imperfect correlate of protection^32,33^; for instance, other immune functions are relevant for protection, while our SVE analysis considers only shifting this single immune marker, consistent with its goal of evaluating the impact of a single immune marker on VE.

*Potential explanation for the overestimation of 3-dose VE predictions against omicron COVID-19*

One potential explanation for why our evaluation of how well SVE estimates could predict 3-dose VE against omicron COVID-19 yielded overestimates is that omicron may have a greater average challenge dose and/or greater viral infectivity^34^ (either/both of which could explain increased transmissibility^35^) compared to pre-omicron viruses^36^, as a greater neutralizing antibody titer is generally needed to protect against greater challenge/infectivity doses.^37,38^

*Differences between Cromer et al. and our study*

Additional differences between Cromer et al.^39^ and our study are that Cromer et al. included 7 studies that assessed VE (including some 2-dose and some 3-dose estimates) against SARS-CoV-2 infection (one study) or against symptomatic COVID-19 (six studies), three of which met our inclusion criteria and were included in our validation analyses. The other four studies used by Cromer et al. were not included in our validation analysis because our inclusion criteria required sequence data confirmation of variants while Cromer et al.’s did not (their analysis allowed genotype inference from epidemiology data). Moreover, our validation analysis included ten studies (nine TND and one RCT) that assessed 2- and/or 3-dose VE against either symptomatic COVID-19 and/or SARS-CoV-2 infection that were not included in Cromer et al.’s. The inclusion criteria of our analysis allowed studies that assessed VE against SARS-CoV-2 infection, a difference from Cromer et al.’s inclusion criteria. Therefore, both differences in the included studies as well as differences in modeling approaches could contribute to explaining slightly different results.

With VE shown to wane with time since vaccination (at least partially due to variant shifts), prediction of VE should account for time since vaccination. Cromer et al. modeled time since vaccination more completely than our approach by always computing standardized GMT for specific periods of follow-up post-vaccination, whereas our analysis restricted to the specific period of follow-up of the COVE study and used empirical VE estimates from external studies that provided results for a similar follow-up period.

**Supplementary Table 1.**

| Variant | Mutations in Spike relative to the index virus (NCBI Ref: NC_045512.2) |
| --- | --- |
| D614G | D614G |
| Alpha^1^ | del69-70,del144,N501Y,A570D,D614G,P681H,T716I,S982A,D1118H |
| Alpha/E484K^1^ | del69-70,del144,E484K,N501Y,A570D,D614G,P681H,T716I,S982A,D1118H |
| Beta | L18F,D80A,D215G,del242-244,(R246I,)K417N,E484K,N501Y,D614G,A701V |
| Delta_AY.1^2^ | T19R,T95I,G142D,del156-157,R158G,W258L,K417N,L452R,T478K,D614G,P681R,D950N |
| Delta_AY.2^2^ | T19R,V70F,G142D,del156-157,R158G,A222V,K417N,L452R,T478K,D614G,P681R,D950N |
| Delta_AY.3^2^ | T19R,G142D,del156-157,R158G,L452R,T478K,D614G,P681R,D950N |
| Epsilon | S13I,W152C,L452R,D614G |
| Gamma | L18F,T20N,P26S,D138Y,R190S,K417T,E484K,N501Y,D614G,H655Y,T1027I |
| Iota | L5F,T95I,D253G,E484K,D614G,A701V |
| Kappa | G142D,E154K,L452R,E484Q,D614G,P681R,Q1071H |
| Lambda | G75V,T76I,R246N,S247-,Y248-,L249-,T250-,P251-,G252-,D253-,L452Q,F490S,D614G,T859N |
| Mu | T95I,insert143T,Y144S,Y145N,R346K,E484K,N501Y,P681H,D950N |
| Omicron_BA.1 | A67V,del69-70,T95I,G142D,del143-145,del211,L212I,+214EPE,G339D,S371L,S373P,S375F,K417N,N440K,G446S,S477N,T478K,E484A,Q493R,G496S,Q498R,N501Y,Y505H,T547K,D614G,H655Y,N679K,P681H,N764K,D796Y,N856K,Q954H,N969K,L981F |
| Omicron_BA.2 | T19I,L24S,P25-,P26-,A27-,G142D,V213G,G339D,S371F,S373P,S375F,T376A,D405N,R408S,K417N,N440K,S477N,T478K,E484A,Q493R,Q498R,N501Y,Y505H,D614G,H655Y,N679K,P681H,N764K,D796Y,Q954H,N969K |
| Omicron_BA.2.12.1 | T19I,L24S,P25-,P26-,A27-,G142D,V213G,G339D,S371F,S373P,S375F,T376A,D405N,R408S,K417N,N440K,L452Q,S477N,T478K,E484A,Q493R,Q498R,N501Y,Y505H,D614G,H655Y,N679K,P681H,S704L,N764K,D796Y,Q954H,N969K |
| Omicron_BA.3 | A67V,H69-,V70-,T95I,G142D,V143-,Y144-,Y145-,N211-,L212I,G339D,S371F,S373P,S375F,D405N,K417N,N440K,G446S,S477N,T478K,E484A,Q493R,Q498R,N501Y,Y505H,D614G,H655Y,N679K,P681H,N764K,D796Y,Q954H,N969K |
| Omicron_BA.4/BA.5 | T19I,L24S,P25-,P26-,A27-,H69-,V70-,G142D,V213G,G339D,S371F,S373P,S375F,T376A,D405N,R408S,K417N,N440K,L452R,S477N,T478K,E484A,F486V,Q498R,N501Y,Y505H,D614G,H655Y,N679K,P681H,N764K,D796Y,Q954H,N969K |

^1^ Data were combined to determine a single geometric mean titer shift for alpha in Figure 2A.

^2^ Data were combined to determine a single geometric mean titer shift for delta in Figure 2A.

**Supplementary Table 2.** Summary statistics of geometric mean pseudovirus neutralizing antibody titers (TZM-bl target cell assay) obtained from participants in clinical studies of the mRNA-1273 vaccine.

|  | | | **Prop (n > LLOD)** | | | **GM (IU50/ml)** | | | **GM (vs. D614G)^1^** | | |
| --- | --- | --- | --- | --- | --- | --- | --- | --- | --- | --- | --- |
| Variant | mRNA-1273 Doses | n | LCL | Est | UCL | LCL | Est | UCL | LCL | Est | UCL |
| D614G | 3 | 26 | 0.87 | 1.00 | 1.00 | 470 | 1371 | 4005 | 1.00 | 1.00 | 1.00 |
| D614G | 2 | 58 | 0.94 | 1.00 | 1.00 | 55 | 462 | 3859 | 1.00 | 1.00 | 1.00 |
| Alpha^2^ | 2 | 28 | 0.88 | 1.00 | 1.00 | 52 | 241 | 1117 | 0.24 | 0.81 | 2.73 |
| Alpha E484K^2^ | 2 | 10 | 0.69 | 1.00 | 1.00 | 16 | 53 | 176 | 0.09 | 0.18 | 0.34 |
| Beta | 3 | 10 | 0.69 | 1.00 | 1.00 | 142 | 784 | 4330 | 0.07 | 0.41 | 2.50 |
| Beta | 2 | 56 | 0.94 | 1.00 | 1.00 | 4 | 43 | 451 | 0.01 | 0.09 | 0.59 |
| Delta AY.1^3^ | 2 | 10 | 0.69 | 1.00 | 1.00 | 24 | 110 | 516 | 0.12 | 0.37 | 1.13 |
| Delta AY.2^3^ | 2 | 10 | 0.69 | 1.00 | 1.00 | 7 | 88 | 1076 | 0.05 | 0.30 | 1.87 |
| Delta AY.3^3^ | 2 | 28 | 0.88 | 1.00 | 1.00 | 26 | 144 | 797 | 0.13 | 0.49 | 1.83 |
| Epsilon | 2 | 28 | 0.88 | 1.00 | 1.00 | 28 | 119 | 509 | 0.19 | 0.40 | 0.86 |
| Gamma | 2 | 25 | 0.86 | 1.00 | 1.00 | 12 | 64 | 332 | 0.08 | 0.23 | 0.64 |
| Iota | 2 | 28 | 0.88 | 1.00 | 1.00 | 20 | 64 | 208 | 0.10 | 0.22 | 0.45 |
| Kappa | 2 | 28 | 0.88 | 1.00 | 1.00 | 12 | 43 | 154 | 0.06 | 0.14 | 0.36 |
| Lambda | 2 | 10 | 0.69 | 1.00 | 1.00 | 61 | 148 | 361 | 0.28 | 0.50 | 0.88 |
| Mu | 2 | 10 | 0.69 | 1.00 | 1.00 | 10 | 32 | 97 | 0.06 | 0.11 | 0.19 |
| Omicron BA.1 | 3 | 10 | 0.69 | 1.00 | 1.00 | 170 | 485 | 1382 | 0.08 | 0.25 | 0.77 |
| Omicron BA.1 | 2 | 30 | 0.54 | 0.73 | 0.88 | 1 | 15 | 396 | 0.00 | 0.02 | 0.33 |
| Omicron BA.1 | 3 | 16 | 0.79 | 1.00 | 1.00 | 30 | 229 | 1733 | 0.07 | 0.21 | 0.62 |
| Omicron BA.2 | 3 | 16 | 0.79 | 1.00 | 1.00 | 31 | 189 | 1158 | 0.06 | 0.17 | 0.49 |
| Omicron BA.2.12.1 | 3 | 16 | 0.79 | 1.00 | 1.00 | 19 | 152 | 1187 | 0.04 | 0.14 | 0.52 |
| Omicron BA.3 | 3 | 16 | 0.79 | 1.00 | 1.00 | 35 | 185 | 967 | 0.07 | 0.17 | 0.38 |
| Omicron BA.4/5 | 3 | 16 | 0.79 | 1.00 | 1.00 | 14 | 92 | 600 | 0.02 | 0.08 | 0.30 |

Table 1 lists the clinical studies in which the vaccine recipients participated. GM, geometric mean; LLOD, lower limit of detection; LCL, 95% lower confidence limit; UCL, 95% upper confidence limit.

^1^ Geometric mean ratios vs. the D614G strain were calculated using the 2-dose or 3-dose D614G result, as appropriate.

^2^ Data were combined to determine a single geometric mean titer shift for alpha in Figure 2A.

^3^ Data were combined to determine a single geometric mean titer shift for delta in Figure 2A.

**Supplementary References**

1 Addetia, A., Crawford, K. H. D., Dingens, A., Zhu, H., Roychoudhury, P., Huang, M. L., Jerome, K. R., Bloom, J. D. & Greninger, A. L. Neutralizing Antibodies Correlate with Protection from SARS-CoV-2 in Humans during a Fishery Vessel Outbreak with a High Attack Rate. *J Clin Microbiol* **58**, doi:10.1128/JCM.02107-20 (2020).

2 Corbett, K. S., Nason, M. C., Flach, B., Gagne, M., O'Connell, S., Johnston, T. S., Shah, S. N., Edara, V. V., Floyd, K., Lai, L., McDanal, C., Francica, J. R., Flynn, B., Wu, K., Choi, A., Koch, M., Abiona, O. M., Werner, A. P., Moliva, J. I., Andrew, S. F., Donaldson, M. M., Fintzi, J., Flebbe, D. R., Lamb, E., Noe, A. T., Nurmukhambetova, S. T., Provost, S. J., Cook, A., Dodson, A., Faudree, A., Greenhouse, J., Kar, S., Pessaint, L., Porto, M., Steingrebe, K., Valentin, D., Zouantcha, S., Bock, K. W., Minai, M., Nagata, B. M., van de Wetering, R., Boyoglu-Barnum, S., Leung, K., Shi, W., Yang, E. S., Zhang, Y., Todd, J. M., Wang, L., Alvarado, G. S., Andersen, H., Foulds, K. E., Edwards, D. K., Mascola, J. R., Moore, I. N., Lewis, M. G., Carfi, A., Montefiori, D., Suthar, M. S., McDermott, A., Roederer, M., Sullivan, N. J., Douek, D. C., Graham, B. S. & Seder, R. A. Immune correlates of protection by mRNA-1273 vaccine against SARS-CoV-2 in nonhuman primates. *Science* **373**, eabj0299, doi:10.1126/science.abj0299 (2021).

3 McMahan, K., Yu, J., Mercado, N. B., Loos, C., Tostanoski, L. H., Chandrashekar, A., Liu, J., Peter, L., Atyeo, C., Zhu, A., Bondzie, E. A., Dagotto, G., Gebre, M. S., Jacob-Dolan, C., Li, Z., Nampanya, F., Patel, S., Pessaint, L., Van Ry, A., Blade, K., Yalley-Ogunro, J., Cabus, M., Brown, R., Cook, A., Teow, E., Andersen, H., Lewis, M. G., Lauffenburger, D. A., Alter, G. & Barouch, D. H. Correlates of protection against SARS-CoV-2 in rhesus macaques. *Nature* **590**, 630-634, doi:10.1038/s41586-020-03041-6 (2021).

4 He, X., Chandrashekar, A., Zahn, R., Wegmann, F., Yu, J., Mercado, N. B., McMahan, K., Martinot, A. J., Piedra-Mora, C., Beecy, S., Ducat, S., Chamanza, R., Huber, S. R., van Heerden, M., van der Fits, L., Borducchi, E. N., Lifton, M., Liu, J., Nampanya, F., Patel, S., Peter, L., Tostanoski, L. H., Pessaint, L., Van Ry, A., Finneyfrock, B., Velasco, J., Teow, E., Brown, R., Cook, A., Andersen, H., Lewis, M. G., Schuitemaker, H. & Barouch, D. H. Low-dose Ad26.COV2.S protection against SARS-CoV-2 challenge in rhesus macaques. *Cell* **184**, 3467-3473.e3411, doi:10.1016/j.cell.2021.05.040 (2021).

5 O'Brien, M. P., Forleo-Neto, E., Musser, B. J., Isa, F., Chan, K. C., Sarkar, N., Bar, K. J., Barnabas, R. V., Barouch, D. H., Cohen, M. S., Hurt, C. B., Burwen, D. R., Marovich, M. A., Hou, P., Heirman, I., Davis, J. D., Turner, K. C., Ramesh, D., Mahmood, A., Hooper, A. T., Hamilton, J. D., Kim, Y., Purcell, L. A., Baum, A., Kyratsous, C. A., Krainson, J., Perez-Perez, R., Mohseni, R., Kowal, B., DiCioccio, A. T., Stahl, N., Lipsich, L., Braunstein, N., Herman, G., Yancopoulos, G. D., Weinreich, D. M. & Covid-19 Phase 3 Prevention Trial, T. Subcutaneous REGEN-COV Antibody Combination to Prevent Covid-19. *N Engl J Med* **385**, 1184-1195, doi:10.1056/NEJMoa2109682 (2021).

6 Benkeser, D., Fong, Y., Janes, H. E., Kelly, E. J., Hirsch, I., Sproule, S., Stanley, A. M., Maaske, J., Villafana, T., Houchens, C. R., Martins, K., Jayashankar, L., Castellino, F., Ayala, V., Petropoulos, C. J., Leith, A., Haugaard, D., Webb, B., Lu, Y., Yu, C., Borate, B., van der Laan, L. W. P., Hejazi, N. S., Carpp, L. N., Randhawa, A. K., Andrasik, M. P., Kublin, J. G., Isaacs, M. B., Makhene, M., Tong, T., Robb, M. L., Corey, L., Neuzil, K. M., Follmann, D., Hoffman, C., Falsey, A. R., Sobieszczyk, M., Koup, R. A., Donis, R. O., Gilbert, P. B., AstraZeneca, A. Z. D. C. S. G., Immune Assays, T. & United States Government /Co, V. P. N. B. T. Immune correlates analysis of a phase 3 trial of the AZD1222 (ChAdOx1 nCoV-19) vaccine. *NPJ Vaccines* **8**, 36, doi:10.1038/s41541-023-00630-0 (2023).

7 Benkeser, D., Montefiori, D. C., McDermott, A. B., Fong, Y., Janes, H. E., Deng, W., Zhou, H., Houchens, C. R., Martins, K., Jayashankar, L., Castellino, F., Flach, B., Lin, B. C., O'Connell, S., McDanal, C., Eaton, A., Sarzotti-Kelsoe, M., Lu, Y., Yu, C., Borate, B., van der Laan, L. W. P., Hejazi, N. S., Kenny, A., Carone, M., Williamson, B. D., Garver, J., Altonen, E., Rudge, T., Huynh, C., Miller, J., El Sahly, H. M., Baden, L. R., Frey, S., Malkin, E., Spector, S. A., Andrasik, M. P., Kublin, J. G., Corey, L., Neuzil, K., Carpp, L. N., Pajon, R., Follmann, D., Donis, R. O., Koup, R. A., Gilbert, P. B., The Immune Assays Team, The Moderna, I. T., The Coronavirus Vaccine Prevention Network (CoVPN)/Coronavirus Efficacy (COVE) Team & The United States Government (USG)/CoVPN Biostatistics Team. Comparing antibody assays as correlates of protection against COVID-19 in the COVE mRNA-1273 vaccine efficacy trial *Science Translational Medicine* **15**, eade9078 (2023).

8 Fong, Y., McDermott, A. B., Benkeser, D., Roels, S., Stieh, D. J., Vandebosch, A., Gars, M. L., Van Roey, G. A., Houchens, C. R., Martins, K., Jayashankar, L., Castellino, F., Amoa-Awua, O., Basappa, M., Flach, B., Lin, B. C., Moore, C., Naisan, M., Naqvi, M., Narpala, S., O’Connell, S., Mueller, A., Serebryannyy, L., Castro, M., Wang, J., Petropoulos, C. J., Luedtke, A., Hyrien, O., Lu, Y., Yu, C., Borate, B., van der Laan, L. W. P., Hejazi, N. S., Kenny, A., Carone, M., Wolfe, D. N., Sadoff, J., Gray, G. E., Grinsztejn, B., Goepfert, P. A., Little, S. J., Paiva de Sousa, L., Maboa, R., Randhawa, A. K., Andrasik, M. P., Hendriks, J., Truyers, C., Struyf, F., Schuitemaker, H., Douoguih, M., Kublin, J. G., Corey, L., Neuzil, K. M., Carpp, L. N., Follmann, D., Gilbert, P. B., Koup, R. A., Donis, R. O., Team, t. I. A., Team, t. J., Team, t. C. V. P. N. E. & Team, t. U. S. G. C. B. Immune Correlates Analysis of a Single Ad26.COV2.S Dose in the ENSEMBLE COVID-19 Vaccine Efficacy Clinical Trial [Preprint] Posted 12 Apr, 2022. Access date 23 Apr, 2022. doi.org/10.1101/2022.04.06.22272763. *medRxiv*, doi:10.1101/2022.04.06.22272763.

9 Gilbert, P. B., Fong, Y., Kenny, A. & Carone, M. A Controlled Effects Approach to Assessing Immune Correlates of Protection. kxac024, <https://doi.org/10.1093/biostatistics/kxac24>. *Biostatistics* (2022).

10 Feng, S., Phillips, D. J., White, T., Sayal, H., Aley, P. K., Bibi, S., Dold, C., Fuskova, M., Gilbert, S. C., Hirsch, I., Humphries, H. E., Jepson, B., Kelly, E. J., Plested, E., Shoemaker, K., Thomas, K. M., Vekemans, J., Villafana, T. L., Lambe, T., Pollard, A. J., Voysey, M. & Oxford, C. V. T. G. Correlates of protection against symptomatic and asymptomatic SARS-CoV-2 infection. *Nat Med* **27**, 2032-2040, doi:10.1038/s41591-021-01540-1 (2021).

11 Khoury, D. S., Cromer, D., Reynaldi, A., Schlub, T. E., Wheatley, A. K., Juno, J. A., Subbarao, K., Kent, S. J., Triccas, J. A. & Davenport, M. P. Neutralizing antibody levels are highly predictive of immune protection from symptomatic SARS-CoV-2 infection. *Nat Med* **27**, 1205-1211, doi:10.1038/s41591-021-01377-8 (2021).

12 Earle, K. A., Ambrosino, D. M., Fiore-Gartland, A., Goldblatt, D., Gilbert, P. B., Siber, G. R., Dull, P. & Plotkin, S. A. Evidence for antibody as a protective correlate for COVID-19 vaccines. *Vaccine* **39**, 4423-4428, doi:10.1101/2021.03.17.20200246 (2021).

13 USG COVID-19 Response Team / Coronavirus Prevention Network (CoVPN) Biostatistics Team. USG COVID-19 Response Team / CoVPN Vaccine Efficacy Trial Immune Correlates Statistical Analysis Plan. figshare. Online resource. <https://doi.org/10.6084/m9.figshare.13198595.v13> Last updated 18 Apr, 2022. Access date 10 Jun, 2022. .

14 Hejazi, N. S., van der Laan, M. J., Janes, H. E., Gilbert, P. B. & Benkeser, D. C. Efficient nonparametric inference on the effects of stochastic interventions under two-phase sampling, with applications to vaccine efficacy trials. *Biometrics* **77**, 1241-1253, doi:10.1111/biom.13375 (2021).

15 Ding, P. & VanderWeele, T. J. Sensitivity Analysis Without Assumptions. *Epidemiology* **27**, 368-377, doi:10.1097/EDE.0000000000000457 (2016).

16 VanderWeele, T. J. & Ding, P. Sensitivity Analysis in Observational Research: Introducing the E-Value. *Ann Intern Med* **167**, 268-274, doi:10.7326/M16-2607 (2017).

17 Hernán, M. A. & Robins, J. M. Causal Inference: What If. Boca Raton: Chapman & Hall/CRC. (2023).

18 Neugebauer, R., van der Laan, M. J., Joffe, M. M. & Tager, I. B. Causal inference in longitudinal studies with history-restricted marginal structural models. *Electron J Stat* **1**, 119-154, doi:10.1214/07-EJS050 (2007).

19 Diaz, I. & van der Laan, M. J. Assessing the causal effect of policies: an example using stochastic interventions. *Int J Biostat* **9**, 161-174, doi:10.1515/ijb-2013-0014 (2013).

20 Haneuse, S. & Rotnitzky, A. Estimation of the effect of interventions that modify the received treatment. *Stat Med* **32**, 5260-5277, doi:10.1002/sim.5907 (2013).

21 Chang, S., Liu, H., Wu, J., Xiao, W., Chen, S., Qiu, S., Duan, G., Song, H. & Zhang, R. Effectiveness of BNT162b2 and mRNA-1273 Vaccines against COVID-19 Infection: A Meta-Analysis of Test-Negative Design Studies. *Vaccines (Basel)* **10**, doi:10.3390/vaccines10030469 (2022).

22 US Food and Drug Administration. SARS-CoV-2 viral mutations: impact on COVID-19 tests. <https://www.fda.gov/medical-devices/coronavirus-covid-19-and-medical-devices/sars-cov-2-viral-mutations-impact-covid-19-tests> (content as in 2021).

23 World Health Organization. Enhancing response to Omicron SARS-CoV-2 variant. (content as in 2021).

24 Public Health England. Investigation of novel SARS-CoV-2 variant of concern 202012/01. Technical Briefing 3 (2021).

25 Public Health England. SARS-CoV-2 variants of concern and variants under investigation in England. Technical Briefing 15 (2021).

26 Pajon, R., Paila, Y. D., Girard, B., Dixon, G., Kacena, K., Baden, L. R., El Sahly, H. M., Essink, B., Mullane, K. M., Frank, I., Denhan, D., Kerwin, E., Zhao, X., Ding, B., Deng, W., Tomassini, J. E., Zhou, H., Leav, B., Schodel, F. & Consortium, C. T. Initial analysis of viral dynamics and circulating viral variants during the mRNA-1273 Phase 3 COVE trial. *Nat Med*, doi:10.1038/s41591-022-01679-5 (2022).

27 Hadfield, J., Megill, C., Bell, S. M., Huddleston, J., Potter, B., Callender, C., Sagulenko, P., Bedford, T. & Neher, R. A. Nextstrain: real-time tracking of pathogen evolution. *Bioinformatics* **34**, 4121-4123, doi:10.1093/bioinformatics/bty407 (2018).

28 Anderson, E. J., Rouphael, N. G., Widge, A. T., Jackson, L. A., Roberts, P. C., Makhene, M., Chappell, J. D., Denison, M. R., Stevens, L. J., Pruijssers, A. J., McDermott, A. B., Flach, B., Lin, B. C., Doria-Rose, N. A., O'Dell, S., Schmidt, S. D., Corbett, K. S., Swanson, P. A., 2nd, Padilla, M., Neuzil, K. M., Bennett, H., Leav, B., Makowski, M., Albert, J., Cross, K., Edara, V. V., Floyd, K., Suthar, M. S., Martinez, D. R., Baric, R., Buchanan, W., Luke, C. J., Phadke, V. K., Rostad, C. A., Ledgerwood, J. E., Graham, B. S., Beigel, J. H. & m, R. N. A. S. G. Safety and Immunogenicity of SARS-CoV-2 mRNA-1273 Vaccine in Older Adults. *N Engl J Med* **383**, 2427-2438, doi:10.1056/NEJMoa2028436 (2020).

29 Westreich, D. & Hudgens, M. G. Invited Commentary: Beware the Test-Negative Design. *Am J Epidemiol* **184**, 354-356, doi:10.1093/aje/kww063 (2016).

30 Sridhar, S., Luedtke, A., Langevin, E., Zhu, M., Bonaparte, M., Machabert, T., Savarino, S., Zambrano, B., Moureau, A., Khromava, A., Moodie, Z., Westling, T., Mascarenas, C., Frago, C., Cortes, M., Chansinghakul, D., Noriega, F., Bouckenooghe, A., Chen, J., Ng, S. P., Gilbert, P. B., Gurunathan, S. & DiazGranados, C. A. Effect of Dengue Serostatus on Dengue Vaccine Safety and Efficacy. *N Engl J Med* **379**, 327-340, doi:10.1056/NEJMoa1800820 (2018).

31 Dayan, G. H., Rouphael, N., Walsh, S. R., Chen, A., Grunenberg, N., Allen, M., Antony, J., Asante, K. P., Bhate, A. S., Beresnev, T., Bonaparte, M. I., Ceregido, M. A., Dobrianskyi, D., Fu, B., Grillet, M.-H., Keshtkar-Jahromi, M., Juraska, M., Kee, J. J., Kibuuka, H., Koutsoukos, M., Masotti, R., Michael, N. L., Reynales, H., Robb, M. L., Martínez, S. M. V., Sawe, F., Schuerman, L., Tong, T., Treanor, J., Wartel, T. A., Diazgranados, C. A., Chicz, R. M., Gurunathan, S., Savarino, S., Sridhar, S. & the VAT00008 study team. Efficacy of a bivalent (D614 + B.1.351) SARS-CoV-2 Protein Vaccine. [Preprint] Posted 13 Jan, 2023. Access date 4 Jul, 2023. doi: <https://doi.org/10.1101/2022.12.05.22282933> *medRxiv*, 2022.2012.2005.22282933, doi:10.1101/2022.12.05.22282933.

32 Gilbert, P. B., Montefiori, D. C., McDermott, A. B., Fong, Y., Benkeser, D., Deng, W., Zhou, H., Houchens, C. R., Martins, K., Jayashankar, L., Castellino, F., Flach, B., Lin, B. C., O'Connell, S., McDanal, C., Eaton, A., Sarzotti-Kelsoe, M., Lu, Y., Yu, C., Borate, B., van der Laan, L. W. P., Hejazi, N. S., Huynh, C., Miller, J., El Sahly, H. M., Baden, L. R., Baron, M., De La Cruz, L., Gay, C., Kalams, S., Kelley, C. F., Andrasik, M. P., Kublin, J. G., Corey, L., Neuzil, K. M., Carpp, L. N., Pajon, R., Follmann, D., Donis, R. O., Koup, R. A., Immune Assays Team section, s., Moderna, I. T. s. s., Coronavirus Vaccine Prevention Network /Coronavirus Efficacy Team section, s. & United States Government /Co, V. P. N. B. T. s. s. Immune correlates analysis of the mRNA-1273 COVID-19 vaccine efficacy clinical trial. *Science* **375**, 43-50, doi:10.1126/science.abm3425 (2022).

33 Gilbert, P. B., Donis, R. O., Koup, R. A., Fong, Y., Plotkin, S. A. & Follmann, D. A Covid-19 Milestone Attained - A Correlate of Protection for Vaccines. *N Engl J Med* **387**, 2203-2206, doi:10.1056/NEJMp2211314 (2022).

34 Syed, A. M., Ciling, A., Taha, T. Y., Chen, I. P., Khalid, M. M., Sreekumar, B., Chen, P. Y., Kumar, G. R., Suryawanshi, R., Silva, I., Milbes, B., Kojima, N., Hess, V., Shacreaw, M., Lopez, L., Brobeck, M., Turner, F., Spraggon, L., Tabata, T., Ott, M. & Doudna, J. A. Omicron mutations enhance infectivity and reduce antibody neutralization of SARS-CoV-2 virus-like particles. *Proc Natl Acad Sci U S A* **119**, e2200592119, doi:10.1073/pnas.2200592119 (2022).

35 Jalali, N., Brustad, H. K., Frigessi, A., MacDonald, E. A., Meijerink, H., Feruglio, S. L., Nygard, K. M., Ro, G., Madslien, E. H. & de Blasio, B. F. Increased household transmission and immune escape of the SARS-CoV-2 Omicron compared to Delta variants. *Nat Commun* **13**, 5706, doi:10.1038/s41467-022-33233-9 (2022).

36 Riediker, M., Briceno-Ayala, L., Ichihara, G., Albani, D., Poffet, D., Tsai, D. H., Iff, S. & Monn, C. Higher viral load and infectivity increase risk of aerosol transmission for Delta and Omicron variants of SARS-CoV-2. *Swiss Med Wkly* **152**, w30133, doi:10.4414/smw.2022.w30133 (2022).

37 Plotkin, S. A. Correlates of protection induced by vaccination. *Clin Vaccine Immunol* **17**, 1055-1065, doi:10.1128/CVI.00131-10 (2010).

38 Plotkin, S. A. & Gilbert, P. B. Nomenclature for immune correlates of protection after vaccination. *Clin Infect Dis* **54**, 1615-1617, doi:10.1093/cid/cis238 (2012).

39 Cromer, D., Steain, M., Reynaldi, A., Schlub, T. E., Sasson, S. C., Kent, S. J., Khoury, D. S. & Davenport, M. P. Neutralising antibodies predict protection from severe COVID-19 [Preprint]. <https://doi.org/10.1101/2022.06.09.22275942>. Posted 14 Jun, 2022. Access date 5 Dec, 2022 *medRxiv* (2022).
